# Fine mapping of *TFL*, a major gene regulating fruit length in snake gourd (*Trichosanthes anguina* L)

**DOI:** 10.1186/s12870-024-04952-6

**Published:** 2024-04-16

**Authors:** Qingwei Jiang, Peng Wang, Yuanchao Xu, Bingying Zou, Shishi Huang, Yuancai Wu, Yongqiang Li, Chuan Zhong, Wenjin Yu

**Affiliations:** 1 College of Agriculture, Guangxi University, Nanning, Guangxi 530004 China; 2 Shenzhen Key Laboratory of Agricultural Synthetic Biology, Agricultural Genomics Institute at Shenzhen, Chinese Academy of Agricultural Sciences, Shenzhen, 518120 China; 3 Yulin Normal College, Yulin, Guangxi 537000 China

**Keywords:** Snake gourd, Fruit length, Map-based cloning, Molecular marker-assisted selection, MADS-box

## Abstract

Fruit length is a crucial agronomic trait of snake gourd (*Trichosanthes anguina* L); however, genes associated with fruit length have not been characterised. In this study, F_2_ snake gourd populations were generated by crossing the inbred lines, S_1_ and S_2_ (fruit lengths: 110 and 20 cm, respectively). Subsequently, bulk segregant analysis, sequencing, and fine-mapping were performed on the F_2_ population to identify target genes. Our findings suggest that the fruit length of snake gourd is regulated by a major-effect regulatory gene. Mining of genes regulating fruit length in snake gourd to provide a basis for subsequent selection and breeding of new varieties. Genotype-phenotype association analysis was performed on the segregating F_2_ population comprising 6,000 plants; the results indicate that the target gene is located on Chr4 (61,846,126–61,865,087 bp, 18.9-kb interval), which only carries the annotated candidate gene, *Tan0010544* (designated *TFL*). *TFL* belongs to the MADS-box family, one of the largest transcription factor families. Sequence analysis revealed a non-synonymous mutation of base C to G at position 202 in the coding sequence of *TFL*, resulting in the substitution of amino acid Gln to Glu at position 68 in the protein sequence. Subsequently, an InDel marker was developed to aid the marker-assisted selection of *TFL.* The *TFL* in the expression parents within the same period was analysed using quantitative real-time PCR; the *TFL* expression was significantly higher in short fruits than long fruits. Therefore, *TFL* can be a candidate gene for determining the fruit length in snake gourd. Collectively, these findings improve our understanding of the genetic components associated with fruit length in snake gourds, which could aid the development of enhanced breeding strategies for plant species.

**Supplementary Information:**

The online version contains supplementary material available at 10.1186/s12870-024-04952-6.

## Introduction

Snake gourds (*Trichosanthes anguina* L; 2n = 2x = 22) are diploid, annual woody climbers of the *Trichosanthes* genus and Cucurbitaceae family [1]. Originating in tropical Asia [2], they are an important ornamental and edible Cucurbitaceae plant. The snake gourd fruit is rich in nutrients, including vitamins, flavonoids, carotenoids, lycopene, and phenolic acids. Ingestion of snake gourds can enhance appetite and nourishment and is beneficial for people with high blood pressure and heart disease [1, 3]. Therefore, the length of the fruit is an important ornamental horticultural trait. However, the genetic and molecular regulatory mechanisms associated with snake gourd length remain unclear, which limits the development of optimal breeding strategies for this plant.

Nevertheless, notable progress has been made in elucidating the regulatory pathways for fruit length in other members of the Cucurbitaceae family, especially in cucumbers, for which 20 quantitative trait loci (QTL) regulating fruit length and shape have been defined [4–6]. Weng et al. [7] mapped nine fruit size-related traits associated with cucumber using three QTL models. The MADS-box transcription factor gene, of which *CsFUL1*^*A*^ is a functional allele, regulates fruit length along with *SF1*, *SF2*, *SF3*, *CsCRC*^*G*^, and *CsaV3_1G044310.* Furthermore, *CsFUL1*^*A*^ inhibits auxin accumulation and cell division or expansion during fruit development by suppressing the expression of its downstream target, *SUPERMAN* (*CsSUP*), and auxin transporter genes, *PIN-FORMED1* (*CsPIN1*) and *CsPIN7*, thus negatively regulating fruit elongation [8]. Meanwhile, the cucurbit-specific RING-type E3 ligase encoded by *SF1* regulates ethylene expression to control cucumber fruit length [9] and promotes cell proliferation by facilitating biological processes and production of the histone deacetylase complex 1 protein encoded by *SF2* [10]. *CsaV3_1G044310* may affect type II inositol polyphosphate 5-phosphate, leading to the distribution and polar transport of auxin and thus affecting auxin-related cell expansion [11]. Small fruits are produced in response to a non-synonymous mutation in SF3—a homolog of the katanin β subunit gene, *KTN1*—reducing cell number [12, 13]. Meanwhile, the homolog of the Arabidopsis crab *CLAW* (*CsCRC*) gene, *CsCRC*^*G*^, on FS5.2 of cucumber positively regulates cell growth and fruit elongation by affecting transcriptional activation of the downstream target gene, *CsARP1* [14, 15].

Additional gene functions have been reported for other members of the Cucurbitaceae family; for example, *BFS* on chromosome 2 in the wax gourd controls fruit shape. BFS is a member of the IQ67-domain protein family that controls Ca^2+^/calmodulin-dependent protein kinase II signal transduction from the cell membrane to the nucleus, thus affecting cell division and cytoskeletal rearrangement to control the fruit shape of wax gourds [16]. There are 159-bp deletions of the *Cla011257* gene that belong to the IQD protein family, affecting the fruit shape in watermelons and leading to fruit elongation [17]. Therefore, in contrast to the genetic mechanisms responsible for regulating fruit shape in watermelons and wax gourds, most genes controlling fruit length in cucumbers indirectly affect the auxin-related response mechanisms of downstream target cells, effectively influencing cell number and size.

One of the largest transcription factor families in eukaryotes is the MADS-box, of which AtAGL6 is a member. AtAGL6 plays an important role in regulating flowering transformation, floral organ formation, and lateral organ development in Arabidopsis [18]. Furthermore, the MADS-box gene, *FRUITFULL* (*FUL*), downregulates the expression of *IND*, *ALC*, *SHP1*, and *SHP2* at the valve margin, thus controlling valve elongation and fruit opening [19–21], resulting in fruits with reduced sizes resistant to cracking. *CiMADS9* overexpression delays flowering and increases the number of leaves in Arabidopsis [22]. Moreover, *ANR1*, an essential gene for the development of root plasticity in *Arabidopsis thaliana*, can mediate root growth in response to NO_3_ availability [23]. Meanwhile, mutations in *ZAG3* alter the phenotypic diversity of male and female flowers in maize [24, 25]. Transcription factors are widely expressed in various eukaryotes and play a vital role in various stages of plant growth and development, including but not limited to flower organ formation, sex differentiation, fruit development, and nutritional growth stages.

The recently published genome assembly of the snake gourd, comprising 202 contigs with a total size of 919.8 Mb and an N50 size of 20.1 Mb [26], has facilitated more in-depth research on the characterisation of genes related to fruit growth. Consumers have different demands for snake gourd fruit length, and our cloning of this gene provides a basis for diversity and molecular selection for snake gourd fruit length and can shorten the breeding cycle.

In this study, the major-effect regulatory gene of snake gourd fruit length was characterised through bulked segregant analysis (BSA), and functional molecular markers were identified to improve the molecular breeding efficiency of new snake gourd varieties with improved fruit length. Elucidating the genetic regulation mechanism and identifying critical genes responsible for fruit length in snake gourds could significantly contribute to the development of new Cucurbitaceae fruit varieties with desirable traits for cultivation and consumption.

## Materials and methods

### Planting materials and phenotypic studies

**Fig. 1:**
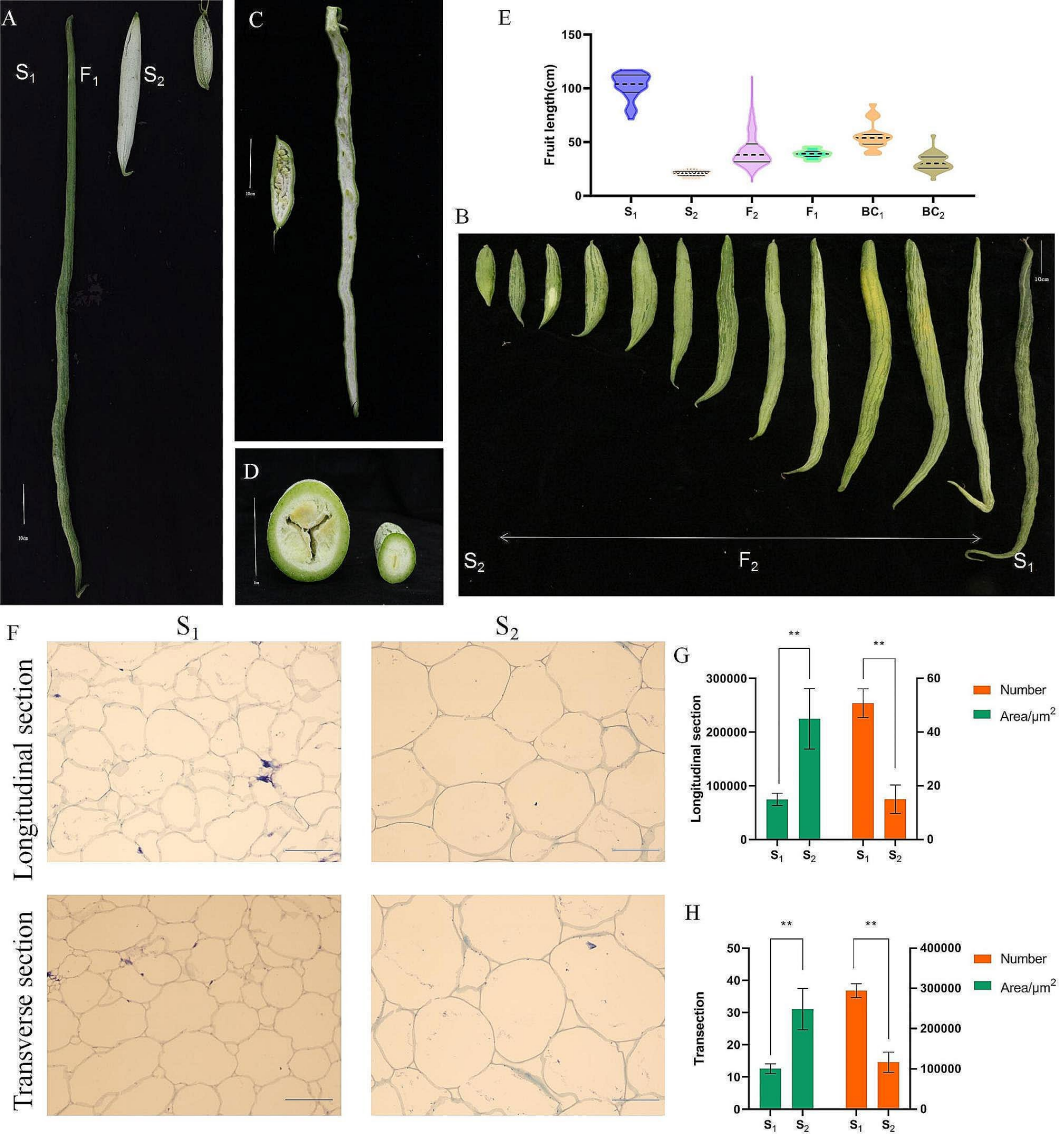
Phenotypic differences in snake gourd length. **(A)** Fruit length variations in S_1_, S_2_, and F_1_; scale bar = 10 cm. **(B)** Fruit length distribution in F_2_; scale bar = 10 cm. **(C)** Vertical section of commodity period fruit (18 days post-pollination) of S_1_ and S_2_; scale bar = 10 cm. **(D)** Cross section of commodity period fruit of S_1_ and S_2_; scale bar = 10 cm. **(E)** Distribution map of six generation lineages: S_1_ (*n* = 30), S_2_ (*n* = 30), F_2_ (*n* = 6,000), F_1_ (*n* = 50), BC_1_P_1_ (*n* = 50), BC_2_P_2_ (*n* = 50). **(F)** Microscopic images of longitudinal and transverse sections of fresh fruit from S_1_ and S_2_ 18 days post-pollination; scale bar = 100 μm. Comparison of **(G)** longitudinal and **(H)** transverse sections of the average cell area and number between S_1_ and S_2_ 18 days post-pollination

Four generating mapping populations like F_1_, F_2_, BC_1_P_1_, and BC_1_P_2_ in snake gourd, two contrasting inbred lines for fruit length such as S_1_ (fruit length: 110 cm, standard deviation: 19.5) and S_2_ (fruit length: 20 cm, standard deviation: 2.7) were used for crossing in this study. In September 2020, the F_1_ population was obtained by crossing S_2_ and S_1_ as female and male parents, respectively, at the vegetable base of Guangxi University (Nanning, China); F_2_ was obtained by self-crossing the F_1_ population in April 2021. In total, 6,000 F_2_ plants were planted in the Shajing test base (longitude: 108°51′E; latitude: 22°48′N) in Nanning, Guangxi, between September 2021 and April 2022. The within-row spacing was 0.5 m, and a 1.2-m gap was left between rows. Three well-developed fruits were retained in each plant to ensure full fruit development. Fruit length was measured with a ruler 40 days after pollination; the mean length of the three fruits was recorded as the length phenotype for each plant. For simplicity, the division of fruit length was based on the phenotypes of the parents and F_1_. The long-fruited phenotypes of the parents range from around 70–110 cm; the short-fruited parents range from around 17–22 cm; and the F1 fruit lengths range from 35.0 to 45.0 cm, with an average of 40.0 cm. We therefore classified the long fruits, as greater than 50 cm and the short fruits as less than 30 cm (Fig. 1A).

### Cytological analysis

The mesocarp from the middle portion of the fruit was cut into thin slices and immediately placed in a formaldehyde/alcohol/acetic acid solution (50% ethanol:40% formaldehyde: glacial acetic acid = 16:1:1) in a 50-mL vial to compare the cytological properties of fruits from the two parental lines on days 9, 6, and 3 before flowering and on days 0, 6, 12, and 18 after pollination. Thin sections were paraffinised and sliced with a microtome into vertical and horizontal sections. Subsequently, the sections were deparaffinised with xylene and treated with a Safranin solid green double stain. The slides were visualised with a Z2 automatic upright differential interference fluorescence microscope (Zeiss, Germany). The cell number (X), cell area (A), and average cell area in a given section were calculated. The area of the whole-fruit longitudinal section (A′) was determined by measuring the ovary or fruit diameter and using the equation for the area of an ellipse (for a longitudinal section). The cell number in the whole-fruit cross sections or longitudinal sections (X′) was calculated using the equation X/A = X′/A′. Cell sizes and number of parental lines were quantified using Image J software (https://imagej.nih.gov/ij/https://imagej.nih.gov/ij/) and evaluated using Microsoft Office Excel 2019 (Microsoft, Redmond, WSP, USA) (Fig. 1C, D).

### DNA extraction

The cetyltrimethylammonium bromide method [27] was used to extract genomic DNA from the leaves of the parent plants and the F_1_ and F_2_ populations. A k5800 ultra-micro spectrophotometer (Kaiao, Beijing, China) was used to measure the concentration and purity of the extracted DNA, and 1.5% agarose gel electrophoresis was used to assess DNA quality.

### BSA sequencing mapping strategy

Of the 2,548 F_2_ plants created by crossing S_1_ and S_2_, 60 with extreme traits (30 long and 30 short) were selected to create two groups with notably opposing phenotypes, the phenotypes of Long and short fruit pool plants is shown in Supplementary Table S1 and S2. After filtering, two pools of extreme trait data were admixed, with one long pool and one short pool being constructed. The two mixed and two-parent pools were used for association analyses. DNA was extracted from young leaves, fragmented by ultrasonication, and subjected to fragment purification and end repair, where adenosine was added at the 3′-end, and sequencing junction ligation was performed. The DNA fragments were then separated via agarose gel electrophoresis to facilitate fragment size selection. Next, the DNA sequences were amplified using PCR to form the sequencing library. The libraries were then sequenced using the Illumina sequencing platform, and raw image data files obtained were transformed into Sequenced Reads after Base Calling analysis. The raw reads were filtered to obtain clean reads, which were used for subsequent analysis. The main data filtering steps included (1) removing reads with adapters; (2) filtering reads with an N content > 10%; (3) removing reads with a mass value < 10 bases and > 50%. Sequencing reads were compared with the reference genome using BWA software and located to the reference genome for subsequent mutation analysis.

Single-nucleotide polymorphisms (SNPs) and InDels were detected using GATK software. Based on the results of the positioning of clean reads in the reference genome, GATK was used for local realisation and other pre-treatments to ensure the accuracy of SNP detection and for SNP detection to determine the SNP site. The filtering criteria were as follows: SNP loci with multiple (> 2) genotypes, SNP and InDel loci with read support < 5, no polymorphism between parents, and loci missing from a sample. The Euclidean distance (ED) association and Δ SNP-index algorithms were used as association analysis techniques [28, 29].

### Fine-mapping

Based on the BSA-seq data and the distribution and density of the physical InDel locations, we designed seven InDel markers for each 1-Mb interval to further narrow the candidate gene regions. The primers used in this study were designed using Primer Premier 5 (Premier, Canada). Each 12-µL reaction volume contained 2 µL DNA template, 1 µL of each forward and reverse primer (10 μm), 5 µL Master Mix, and 3 µL ddH_2_O. The PCR cycle conditions were as follows: 95 °C for 5 min, 30–35 cycles of 95 °C for 30 s, 50–58 °C for 30 s, 72 °C for 30 s, extension at 72 °C for 5 min, and incubation at 4 °C. The digested and PCR amplification products were separated for 1 h at 300 V on a native 8% polyacrylamide gel. The resulting polyacrylamide gel is presented in Supplementary Table S3. A total of 526 long-fruit and 493 short-fruit plants from 2,548 F_2_ individuals were used for genotype-phenotype analysis. To further narrow down the mapping range, five new InDel markers were applied to genotype the F_2_ population (*n* = 6,000 individuals) and identify recombinants. The genotypes of the recombinant plants and the most likely target gene region were inferred using a genotype-phenotype joint analysis.The primer sequence is shown in Supplementary Table S4.

### Cloning and sequencing analysis of candidate genes

Forward and reverse primers were designed according to the coding sequence (CDS) of the gene (Supplementary Table S4). Tender leaf RNA was extracted, reverse transcribed into cDNA, and amplified with 2× Phanta Max Master Mix (Vazyme, Nanjing, China). Axyprep DNA gel extraction kits (Axygen, Union City, CA, USA) were used for the recovery and purification of target bands from agarose gels. For subsequent experiments, pure DNA extracted from the gels was used.

A Zero Blunt TOPO PCR Cloning Kit (CV16; Aidlab, Beijing, China) was used to construct the recombinant plasmid, which was then cloned in *Escherichia coli DH5α* competent cells. Subsequently, colony PCR and agarose gel electrophoresis was performed to detect positive bacterial colonies; the five bacterial colonies with the brightest DNA bands were sent to Shenggong Biotechnology Co. (Shanghai, China) for sequencing. DNAMAN V.9 (Lynnon Biosoft, CA, United States) was used to compare amino acid and DNA sequences (Supplementary Table S5).

### Analysis of spatiotemporal gene expression

To analyse gene expression in different parts and stages, RNA was extracted from the parent root, stem, and leaf. RNA from the ovary during different development stages (9, 6, and 3 days before flowering and 0, 6, 12, and 18 days after pollination) was extracted using an Eastep Super Total RNA extraction kit (Promega, Shanghai, China) following the manufacturer’s instructions. The primers for histone H3 (Tan0019208.1) and other candidate genes were designed using Primer Premier 5.0. The cDNA was synthesised using reverse transcriptase RT Master Mix (RR036A) following the manufacturer’s instructions (TaKaRa, Beijing, China). Each quantitative reverse transcription PCR (qRT-PCR) mixture was prepared and the quantitative fluorescence was analyzed according to the methods described by Huo et al. [30]. Each quantitative reverse transcription PCR (qRT-PCR) mixture contained 2 µL cDNA, 0.8 µL forward and reverse primers, 10 µL TB Green Premix Ex TaqII, 0.4 µL ROX Reference Dye II, and 6 µL nuclease-free water. Quantitative fluorescence analysis was performed using the Applied Biosystems 7500 real-time PCR instrument (Foster City, CA, USA). Briefly, the samples were preheated at 95 °C for 30 s, followed by heating for 5 s at 95 °C, and 40 cycles at 60 °C for 34 s. High-resolution melting was performed at 95 °C for 15 s, 60 °C for 1 min, and 95 °C for 15 s. Each sample was tested for relative gene expression using the 2^−∆∆Ct^ method for three biological replicates [31] subsequently analyzed using SPSS 25.0.

### Phylogenetic analyses

To understand the relationship between the TFL protein sequence and other homologous sequences, the gene sequence was downloaded in FASTA format from the NCBI database and performed an NCBI BLAST search (NCBI, Bethesda, MD, USA). Subsequently, using the MEGA-X software’s neighbour-joining function, we created a phylogenetic tree with 1,000 replicates (Supplementary Table S6).

### Molecular marker-assisted selection test

Based on the InDel differences of *TFL* in the parents, located in the intron, InDel markers were developed according to candidate gene sequences using Primer Premier 5 software. Beijing Tsingke Biotechnology Co., Ltd. (Nanning, China) synthesised all primers used in this study (Supplementary Table S4). The same steps were followed as those in Sect. 2.5 for the InDel marker generation. Genotype-phenotype analysis was performed for all seed resources tested. Twenty-seven germplasm resources of snake gourd were validated and divided into long-fruited and short-fruited groups (Supplementary Table S7).

## Results

### Phenotypic and genetic characteristics of fruit length

**Fig. 2:**
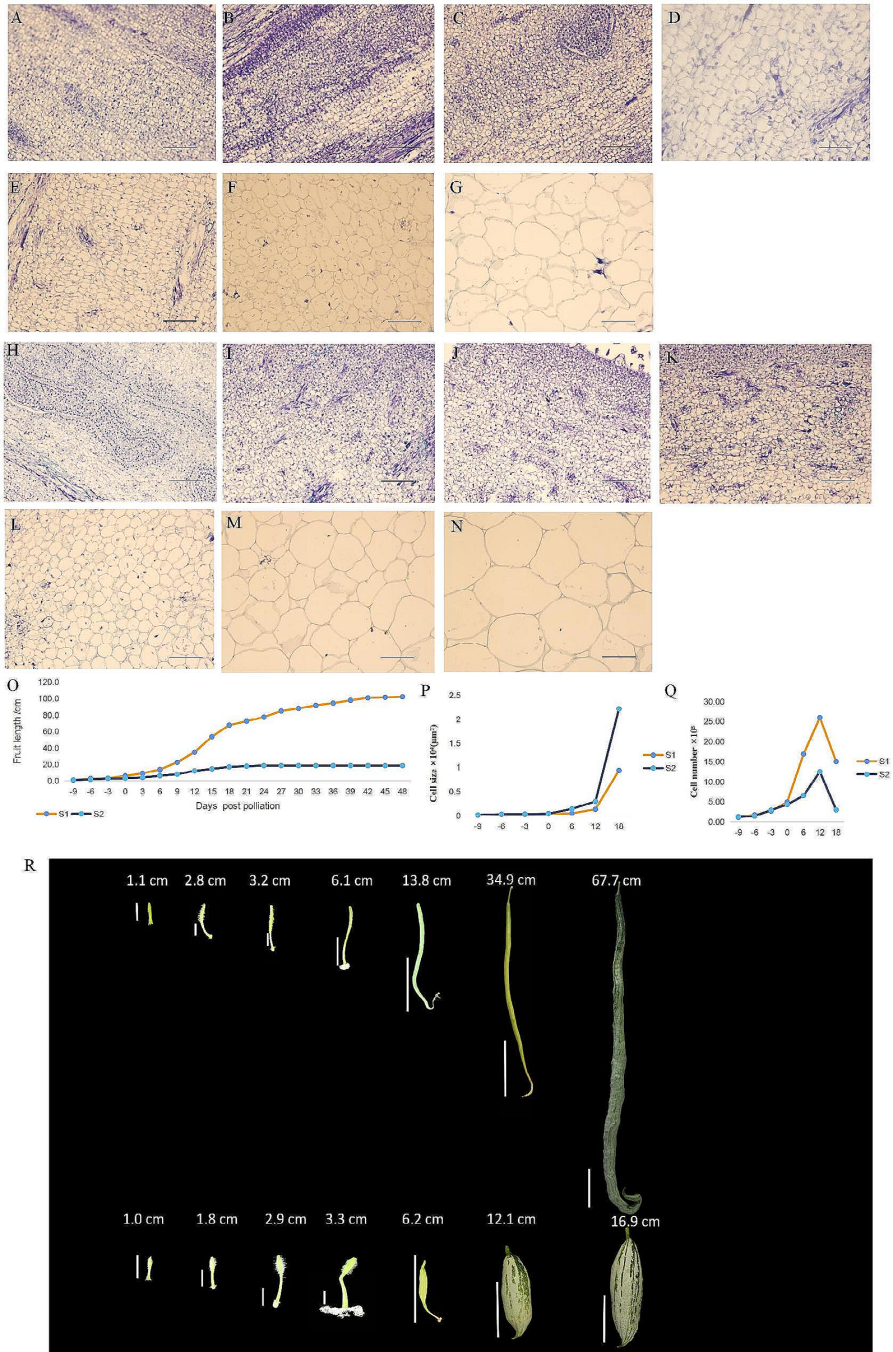
**(A)–(G)** Longitudinal section of the ovary in the S_1_ inbred line on days 9, 6, and 3 before flowering and on days 0, 6, 12, and 18 after pollination (scale bar = 100 μm). **(H)–(N)** Longitudinal section of the ovary in the S_2_ inbred line on days 9, 6, and 3 before flowering and days 0, 6, 12, and 18 after pollination. **(O)** Fruit length growth cycle curve of S_1_ (green) and S_2_ (orange). **(P)** Average cell size during fruit development in S_1_ and S_2_. **(Q)** Cell proliferation during fruit development in S_1_ and S_2_. **(R)** From left to right: days 9, 6, and 3 before flowering and day 0 after pollination (scale bar = 1 cm); days 6, 12, and 18 after pollination (scale bar = 10 cm)

Longitudinal fruit diameter (fruit stalk to receptacle) in the parents was measured 9, 6, and 3 days before flowering and 0, 6, 12, and 18 days after pollination until fruit length was set (Fig. 2R), and the growth curve was plotted (Fig. 2O). BC_1_P_1_ fruit lengths ranged from 38.4 to 85.0 cm with a mean of 55.9 cm and a standard deviation of 12.6. BC_1_P_2_ fruit lengths ranged from 15.0 to 56.0 cm with a mean of 31.2 cm and a standard deviation of 7.5. The rapid-growth period of the snake gourd occurred 6–18 days after pollination; the fruit length in the F_2_ population conformed to a normal distribution (Fig. 1B, E). Fruit lengths of < 30 cm and > 50 cm were classified as short and long, respectively. Of the 6,000 F_2_ plants, 1,189 had short-fruit, and 1,269 had long-fruit. No differences in cell proliferation rate or cell size were observed between S_1_ and S_2_ before pollination. Mostly intermediate, with a relatively small number of extremely long and extremely short fruits. However, differences arose after pollination, with S_1_ having a much higher rate of cell proliferation than S_2_. Meanwhile, the cell area amplification rate was markedly higher in S_2_ than in S_1_. On day 18, S_1_ had seven times more cells than S_2_, and S_2_ had twice the cell area of S_1_. Although the number of cells decreased after 12 days of pollination, the increase in cell area compensated for the quantity. At fruit maturity, the length of S_1_ fruit was five times greater than S_2_ (Figs. 1F-H and 2A-N, P and Q).

### Fine-mapping and identification of the candidate gene

- Whole genome sequencing of bulks and parental lines.

After whole-genome resequencing, 238,457,730, 221,257,766, 234,769,954 and 224,787,160 of Reads numbe were obtained from the S_1_, S_2_ ,and two extremes of mixed pools populations, respectively. The Q30 and GC values for S_1_ are 94% and 38%, respectively, with a size of 35.7 GB, while the corresponding values for S_2_ are 93% and 37%, respectively, with a size of 33.1 GB.The corresponding values for the two extremes of the mixed pool are 93% and 38%, 92% and 38%, respectively, with sizes of 35.2 GB and 33.7 GB. The statistics of sequencing data is in the Supplementary Table 8. Compared with the reference genome, the similarity rate between parents and the F_2_ population was 91.09–91.58%. The average sequencing depth of the parental genome was 33.6×.

(b) QTL identification for fruit length.

Before association analysis, SNPs and InDels were filtered, resulting in the identification of 1,343,961 SNPs and InDel markers. We used the indexing algorithm to analyse the association between SNP markers and selected the region with a threshold > 1.35 and the Δ SNP-index values is 99%. Candidate intervals were calculated using the ED and SNP-index algorithms. Because the SNP-index interval was within the ED algorithm interval, we selected the concatenation interval (59.677,903 bp − 63,802,574 bp, Δ SNP-index value is 0.71).

(c) QTL validation using single marker analysis.

**Fig. 3:**
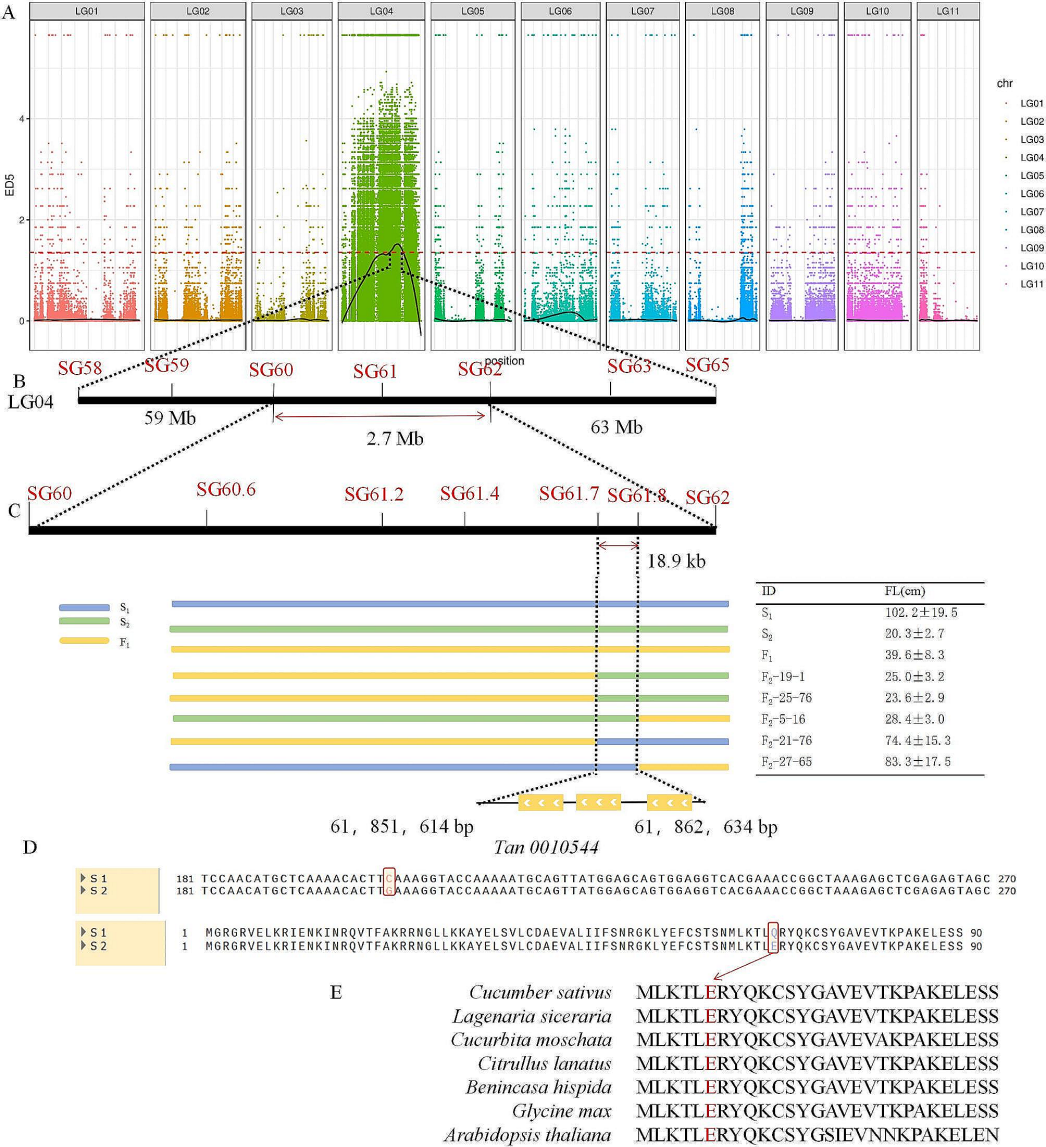
Genetic mapping of the gene regulating fruit length in snake gourds. **(A)** Distribution of the Euclidean distance (ED) association values on chromosomes (chr). The abscissa indicates the chr name, the coloured dots represent the ED value of each single nucleotide polymorphism (SNP) locus, the black line represents the fitted ED value, and the red dotted line represents the significance association threshold. The higher the ED value, the better the association effect of the SNP locus. **(B)** Fine-mapping of the fruit length gene using InDels. Using 2,548 F_2_ individuals resulting from the S_1_ × S_2_ cross, the location of the gene regulating fruit length was narrowed down to a 2.7-M region between the markers, SG60 and SG62, on chr 4. **(C)** Genotyping of recombinant plants from 6,000 F_2_ individuals resulting from the S_1_ × S_2_ cross. The fruit length of the two parents and their F_1_ offspring and the genotype of the recombinant single parent are shown on the right. The location of the gene that regulates the length of the fruit was narrowed down to an 18-kb region. **(D)***Tan0010544* (*TFL*) contains the c.202 C > G mutation in the coding sequence and p.Gln68Glu substitution in the protein sequence. (**E**) Alignment of *Tan0010544* (*TFL*) homologs from different species. The E68Q mutation in S_1_ is highlighted in red

Developed one marker per 1-M interval within the interval. Seven InDel markers were first developed with 526 long-fruit and 493 short-fruit plants from 2,548 F_2_ plants, and a genotype-phenotype association analysis was performed to compress the interval between the SG60 (60,655,536 bp) and SG62 (63,389,272 bp) markers. Five new InDel markers were then developed within this de-interval, the genotype-phenotype of 1,269 long-fruit and 1,189 short-fruit plants from 6000 F_2_ plants were analyzed, and five recombinant plants were identified in the extreme phenotype plants. ID is F_2_-19-1, F_2_-25-76, F_2_-5-16,F_2_-21-76 and F_2_-27-65 (Supplementary Table S9). As shown in Fig. 3A-C, the genotypes of F_2_-19-1 and F_2_-25-76 are shown as short fruits in marker SG61.7, and the genotype of F_2_-5-16 is shown as short fruits in marker SG61.8, and all the three fruit lengths are short fruits. The genotypes of F_2_-21-76 is shown as long fruits in marker SG61.7, and the genotype of F_2_-27-65 is shown as long fruits in marker SG61.8, aand all the two fruit lengths are long fruits. Therefore, the interval was narrowed down to SG61.7 (61,846,126 bp) - SG61.8 (61,865,087 bp), a total of 18.9 kb, by genotype-phenotype association analysis, and only one gene, *Tan0010544* (*TFL*), was found in this region. Therefore, *TFL* was a candidate gene for controlling the fruit length of snake gourds (Fig. 3A–C).

(d) Candidate gene identification.

Candidate gene and their whole CDSs were cloned. To determine the *TFL* sequence, we cloned its CDS from the two parental lines and performed a sequence comparison analysis. The total lengths of *TFL* and its CDS were 11.02 kb and 741 bp, respectively. Sequence analysis revealed a non-synonymous mutation of base C to G at position 202 in the coding sequence of *TFL*, resulting in a substitution of amino acid Gln to Glu at position 68 in the protein sequence (Fig. 3D). Alignment of the S_1_ and S_2_ amino acid sequences with those of homologous proteins from various plant species showed that E–68 is conserved among land plants (Fig. 3E). The *TFL* determines the interval inSupplementary Table 10.

### Gene expression analysis

**Fig. 4:**
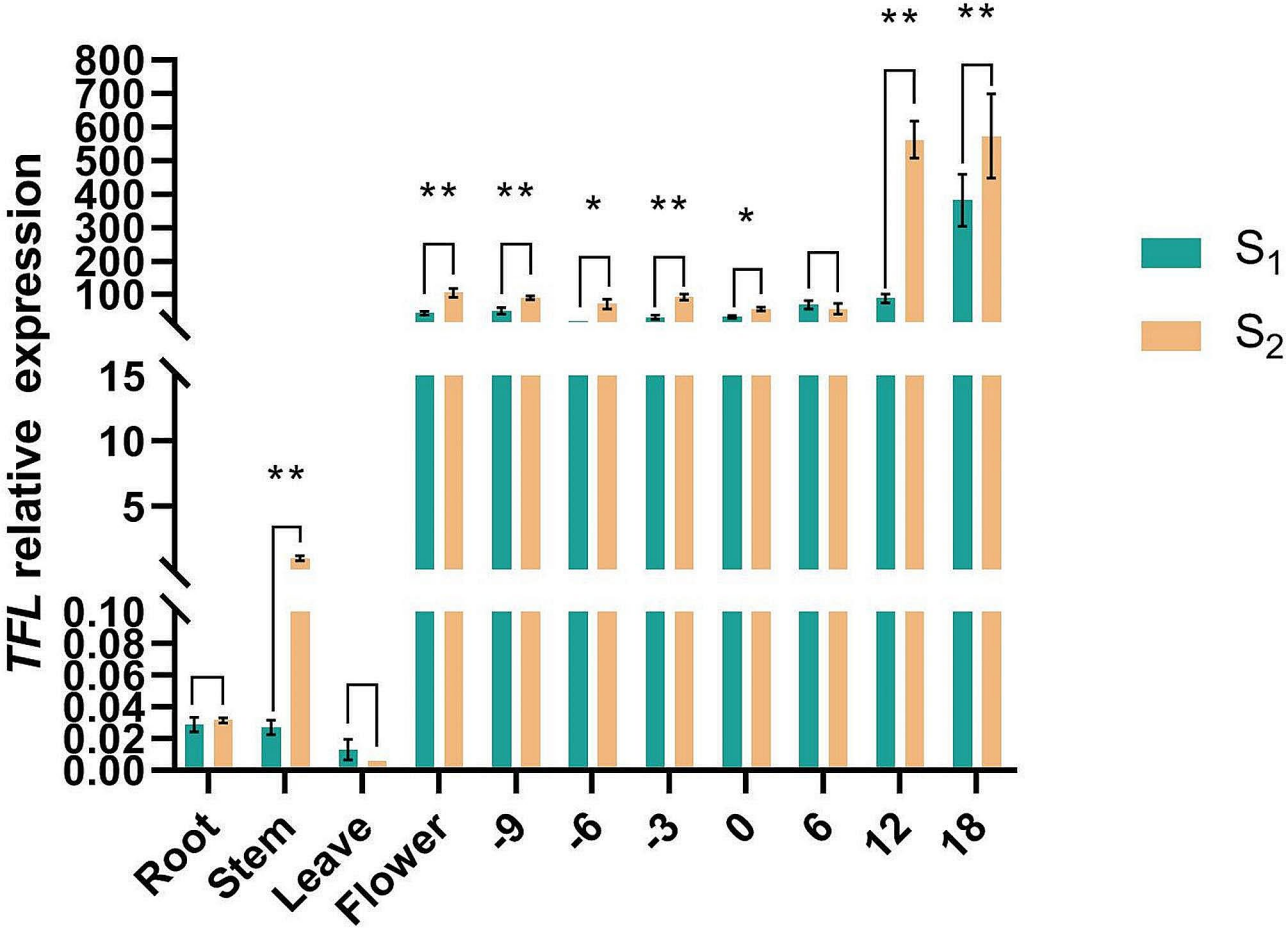
Quantitative real-time PCR analysis to assess the relative expression of *Tan0010544* (*TFL*) in roots, stems, leave and male flowers of the S_1_ and S_2_ inbred lines and at different periods during the ovarian formation, particularly on days 9, 6, and 3 before flowering and days 0, 6, 12, and 18 after pollination using quantitative real-time PCR. *P < 0.05, **P < 0.01

The expression of *TFL* was compared between the root, stem, leaf, and male flower in S_1_ and S_2_. Furthermore, *TFL* expression was examined at different periods during ovary formation, particularly on days 9, 6, and 3 before flowering and days 0, 6, 12, and 18 after pollination. qRT-PCR analysis revealed no difference in *TFL* expression between roots, leaves, and ovary 6 days after pollination. However, significant differences (0.01 < *P <* 0.05) were observed in the ovary 6 days before flowering and 0 days after pollination. The expression of *TFL* in the stem and male flower differed significantly (*P <* 0.01) on day 9 before flowering and on days 12 and 18 after pollination. Comprehensive analysis of paraffin sections and the growth cycle revealed an inverse correlation between cell number and area, with higher *TFL* expression levels corresponding to shorter fruit length and reduced cell numbers, yet larger cell areas (Figs. 2A–O and 4). The expression of *TFL* increased significantly in parents 12 days after pollination, and this led us to suspect that this gene may be related to cell development (Figs. 2P–Q and 4). Meanwhile, the regulatory mechanisms linking *TFL* with fruit length remain unknown.

### Phylogenetic analysis

**Fig. 5:**
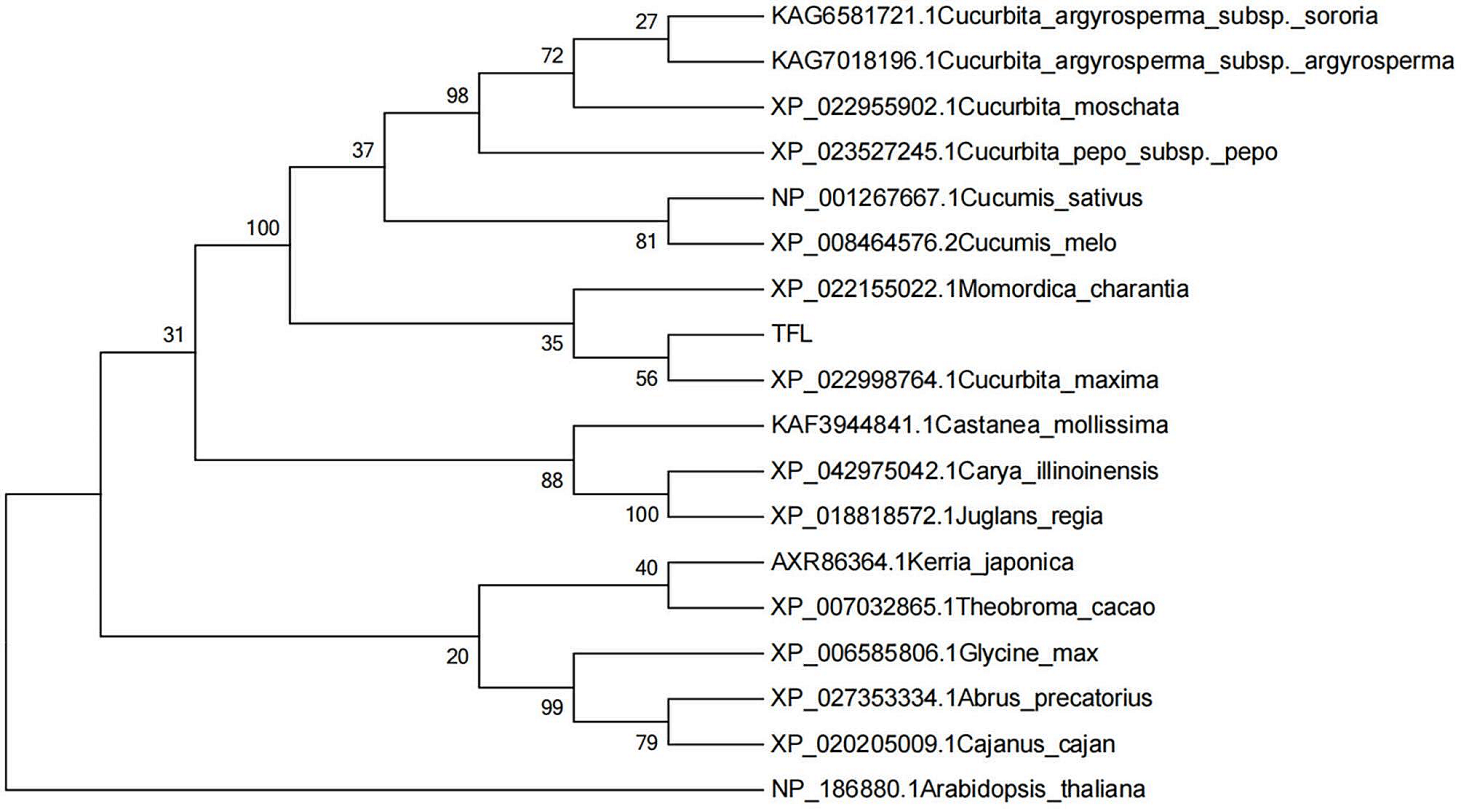
Phylogenetic tree of *Tan0010544* (*TFL*) and its homologous proteins. The phylogenetic tree was constructed using MEGA-X software with 1,000 bootstrap replications. The numbers at the tree nodes indicate bootstrap values

The results of the phylogenetic analysis revealed that *TFL* had a close phylogenetic relationship with legumes and Cucurbitaceae, indicating that TFL was evolutionarily conserved in Cucurbitaceae. Sequence alignment results indicated that *TFL* shares 95% sequence homology with XP_022998764.1 found in *Cucurbita maxima.* (Fig. 5).

### Development of InDel markers for molecular marker-assisted breeding

**Fig. 6:**
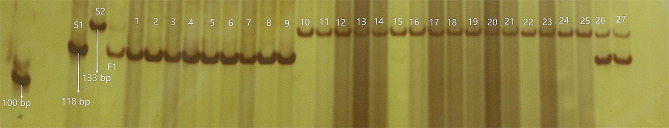
In total, 27 different lines of snake gourd were analysed using InDel markers. The first 9 and last 16 corresponded to long and short fruit parent phenotypes, respectively. We observed inconsistencies between the genotypes and phenotypes of species 26 and 27 in relation to those of their parents

In total, 27 snake gourd inbred lines with extreme fruit length differences, including 9 long and 18 short fruits, were selected for validation using the InDel molecular marker. We observed that the bands of 9 long-fruited snake gourd materials were consistent with S_1_, whereas those of 16 short-fruited snake gourds were consistent with S_2_. However, the bands from samples 26 and 27 were inconsistent with those of F_1_. The correlation rate between genotype and phenotype was 92% (Fig. 6). Collectively, our results suggest that the InDel marker can be used in the molecular marker-assisted breeding of snake gourd fruit length.

## Discussion

### Relationship between fruit length and *TFL* expression in snake gourds

In this study, we used a pair of inbred snake gourd lines with significant differences in fruit lengths as parental strains. A major effect gene affecting the fruit length of the snake gourd was located using the BSA-seq method. Extreme long and extreme short fruits were used for primary localisation, similar in fruit length to the long-fruited parent and short-fruited parent, respectively, to obtain the BSA primary interval. The results showed that the interval was 59,677,903 bp − 63,802,574 bp on chromosome 4. The intervals exceeding 99% of the Δ SNP-index value were selected as the intervals to be finely localised later, and for the sake of the accuracy of the experiment, I discarded the intermediate values of the F2 monocots, and only the phenotypically extreme monocots were used for the fine localisation. We narrowed the interval using extreme phenotypes to 18 kb according to the genotype-phenotype approach and confirmed that *Tan0010544* (*TFL*), a MADS-box family gene, implicates that *TFL* is involved in regulation of fruit length. We also confirmed by qRT-PCR analysis that *TFL* expression does not differ significantly between roots and leaves but is significantly different in stems and flowers. Although the lengths of the ovaries and the cell number and area of the parents 9 days before flowering did not differ significantly, *TFL* expression was markedly different. The expression of *TFL* at 9 days before flowering coincides with the differential growth rate of the ovary, leading to a two-fold difference in fruit length at flowering. The fruit length of S_2_ 0 days after pollination was half that of S_1_. The ovaries underwent rapid growth and development 6 days after pollination. Starting from 9 days pre-pollination to 18 days after pollination, *TFL* expression increased significantly; this was mirrored, subsequently, by a significant change in fruit length. By 12 days post-pollination, the S_1_ and S_2_ fruit length increased five times and 2.5 times, respectively, compared to 0 days after pollination. Additionally, *TFL* expression in S_1_ increased significantly, whereas the growth rate and rate of fruit length growth decreased.

Collectively, the growth cycle curve and gene expression in parents suggest that the *Tan0010544* (*TFL*) gene is associated with the fruit length of snake gourds. We speculate that the *TFL* gene may affect the auxin regulation pathway to regulate fruit growth in snake gourds. Auxin plays a crucial role in cell division and differentiation, as well as in fruit development [32]. Therefore, *TFL* may also affect cell number and area. Although the length of the parents was the same 9 days before flowering, the difference in *TFL* expression corresponded with cell division and differentiation. The paraffin sections did not reveal significant differences in the number or area of S_1_ and S_2_ cells 9 days before flowering, whereas the area of S_2_ cells was two times greater than that of S_1_ during the last 18 days. Meanwhile, there were seven times more S_1_ cells than S_2_ cells. Collectively, the increase in the number of cells in S_1_ than in S_2_ may contribute to the increase in the fruit length of S_1_. Thus, *TFL* could affect cell division and regulate the fruit length of snake gourds.

### Association of *TFL* function with the MADS-box transcription factor family

The MADS-box gene contains a highly conserved region that encodes a MADS-box domain composed of approximately 60 amino acids. Previous phylogenetic analysis of the MADS-box gene in fungi, plants, and animals [33] has revealed that the gene underwent a replication event before differentiation between animals and plants, producing type I (SRF-like) and type II (MEF2-like) lineages. Using the structure of *A. thaliana* for duplication and motif analysis, type I and type II MADS-box genes were further divided into five subtypes: Mα, Mβ, Mγ, Mδ, and MIKC [34]. Phylogenetic analysis of the rice MADS-box gene family revealed that the type I gene contains Mα, Mβ, Mγ, and Mδ subfamilies, whereas type II comprises MIKC subgroups [35]. The MADS-box gene family reportedly encodes many transcription factors that play a vital role in the growth and development of various plants, including fruit growth and development [21], seed colour change, and embryo development [36]. It also contributes to stress resistance, as to well as cell and organ differentiation. There are several reports on the MADS-box family that affect flower development. For example, APETALA1, APETALA3, and PISTILLATA act as bifunctional transcription factors (activating and inhibiting factors) and affect flower patterning and fruit ripening [37–39]. Zhao et al. [8] reported that the MADS-box family gene, *CsFUL1*^*A*^, affects fruit length by regulating auxin, cell number, and size.

In this study, the expression of *TFL* in the flowers and stems of the parents also differed significantly. The MADS-box family affects the growth and development of flowers, and *TFL* expression was reportedly significantly different in floral organs. Smaczniak et al. [40] reported that the MADS-box transcription factor can bind to the CArG-box of the target gene. CsSUP has three CArG boxes in the promoter region, one CArG-box in the coding region, and one type A CArG-box located 2,200 bp from the transcription start site and binds to the MADS-box protein [41]. Zhao et al. [8] confirmed that *CsFUL1*^*A*^ binds to the CArG-box of the promoter region, inhibiting the expression of *SUPERMAN*, a regulator of cell division and expansion. Collectively, we speculate that the *TFL* gene may also combine with the CArG-box region of the *SUPERMAN* promoter and subsequently inhibit the expression of *SUPERMAN* to further inhibit cell division and expansion. This could inhibit the expression of the auxin transporters, *PIN-FORMED1* (*PIN1*) and *PIN7*, both of which jointly control the fruit length of snake gourds, reducing the accumulation of auxin. *TFL* encodes a typical type II MADS-box protein with four modular domains (MIKC), the N-terminal DNA-binding MADS domain (M), the intervening (I) and keratin-like (K) regions, and a C-terminal region (C) (Supplementary Table S11 and Supplementary Fig. 1).

In this study, a major-effect gene (*TFL*) regulating fruit length was cloned for the first time in a pair of snake gourds with significant differences in fruit length using the BSA method combined with genotype-phenotype analysis of F_2_ recombinant plants. Furthermore, a functional molecular marker was developed for molecular marker-assisted selection in snake gourds. Samples 26 and 27 may be affected by other non-alleles, and these two materials also provide a material basis for us to continue to explore other non-alleles of snake gourd fruit length, and we will also carry out the study of non-alleles of snake gourd fruit length in these two materials, so as to provide a theoretical basis for comprehensively analyzing the basis of the molecular regulation of snake gourd fruit length. To our knowledge, this is the first report of a gene related to fruit length in snake gourds. This study provides novel insights into snake gourd rapid breeding and could help improve the efficient selection of new varieties of snake gourd for fruit length orientation. In China, snake gourd is mostly used as an ornamental crop, attracting consumers with its exaggerated fruit length. Moreover, our data provide a theoretical basis for future analyses focused on delineating the underlying mechanisms that regulate fruit length at the genetic level. This may allow for a more comprehensive domestication of the Cucurbitaceae family, and a more controlled manipulation of their traits for agricultural applications.

*TFL* belongs to the MADS-box family, one of the largest transcription factor families. Sequence analysis revealed a non-synonymous mutation of base C to G at position 202 in the coding sequence of *TFL*, resulting in the substitution of amino acid Gln to Glu at position 68 in the protein sequence. We speculate that the TFL gene may affect the auxin regulation pathway to regulate fruit growth in snake gourds. An InDel marker was developed to aid the marker-assisted selection of *TFL.*

### Electronic supplementary material

Below is the link to the electronic supplementary material.

Supplementary Material 1

Supplementary Material 2

## Data Availability

The data that support the findings of this study have been deposited into CNGB Sequence Archive (CNSA) of China National GeneBank DataBase (CNGBdb) with accession number CNP0004134. https://db.cngb.org/search/?q=CNP0004134. The data that supports the findings of this study are available from the corresponding author upon reasonable request.
