# Supplementary figures and images for: Fine mapping of TFL, a major gene regulating fruit length in snake gourd (Trichosanthes anguina L)
Source: BMC Plant Biol. 2024 Apr 16;24:286. doi: 10.1186/s12870-024-04952-6 (PMC11020775; doi:10.1186/s12870-024-04952-6)

Thank you for checking it out, above is my additional material.

1.
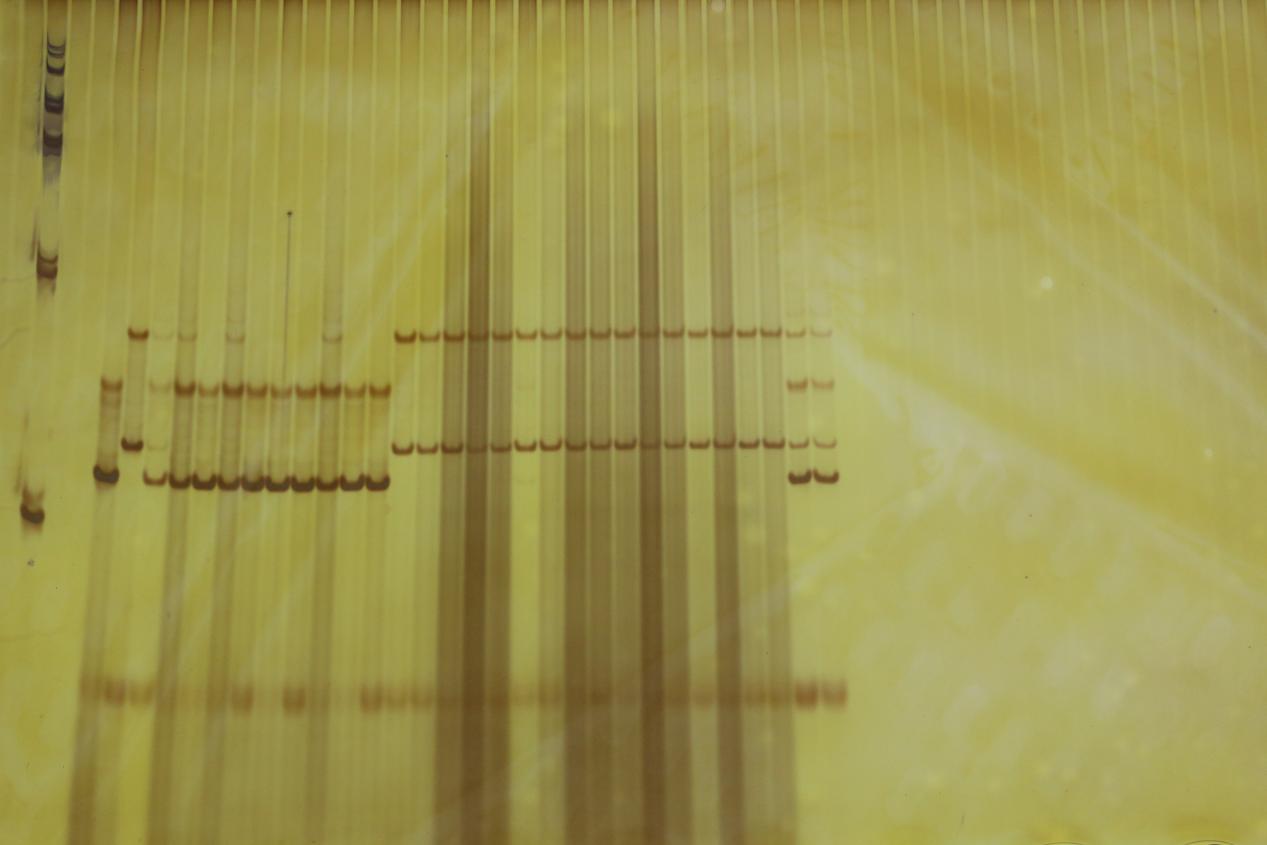

Supplement: Supplementary file 1 — Supplementary Material 1 [file 12870_2024_4952_MOESM1_ESM.docx]
