# Supplementary material for: Fine mapping of TFL, a major gene regulating fruit length in snake gourd (Trichosanthes anguina L)
Source: BMC Plant Biol. 2024 Apr 16;24:286. doi: 10.1186/s12870-024-04952-6 (PMC11020775; doi:10.1186/s12870-024-04952-6)
Supplement: Supplementary file 2 — Supplementary Material 2 [file 12870_2024_4952_MOESM2_ESM.docx]

Supplementary Material

**Fine-mapping the major-effect regulatory genes controlling fruit length in snake gourd (*Trichosanthes anguina* L)**

**Qingwei Jiang^a^, Peng Wang^a^, Yuanchao Xu^b^, YuanCai Wu^a^, Shishi Huang^a^, Jieming Zheng^a^, Yongqiang Li^a^, Huarong Fang^a^,Bingying Zou^a^, Chuan Zhong^a^,Wenjin Yu^a,*^**

***Corresponding author:**

1. **mail address:**yuwjin@gxu.edu.cn

**Table S1** Phenotypes of Short fruit pool plants

| Short fruit pool | FL(cm) |
| --- | --- |
| 1 | 18.0 |
| 2 | 18.4 |
| 3 | 18.5 |
| 4 | 18.6 |
| 5 | 19.0 |
| 6 | 19.2 |
| 7 | 19.8 |
| 8 | 19.9 |
| 9 | 19.9 |
| 10 | 20.1 |
| 11 | 20.1 |
| 12 | 20.2 |
| 13 | 20.3 |
| 14 | 20.5 |
| 15 | 20.5 |
| 16 | 20.9 |
| 17 | 21.0 |
| 18 | 21.1 |
| 19 | 21.1 |
| 20 | 21.2 |
| 21 | 21.4 |
| 22 | 21.8 |
| 23 | 22.0 |
| 24 | 22.0 |
| 25 | 22.0 |
| 26 | 22.1 |
| 27 | 22.1 |
| 28 | 22.2 |
| 29 | 22.2 |
| 30 | 22.2 |
| Average | 20.6 |

**Table S2** Phenotypes of Long fruit pool plants

| Long fruit pool | FL(cm) |
| --- | --- |
| 1 | 111.0 |
| 2 | 103.0 |
| 3 | 100.2 |
| 4 | 103.0 |
| 5 | 100.2 |
| 6 | 98.4 |
| 7 | 96.2 |
| 8 | 93.5 |
| 9 | 92.0 |
| 10 | 90.1 |
| 11 | 89.2 |
| 12 | 88.6 |
| 13 | 87.7 |
| 14 | 86.2 |
| 15 | 86.0 |
| 16 | 85.5 |
| 17 | 84.7 |
| 18 | 84.5 |
| 19 | 82.1 |
| 20 | 82.0 |
| 21 | 82.0 |
| 22 | 81.4 |
| 23 | 81.3 |
| 24 | 81.3 |
| 25 | 81.0 |
| 26 | 80.6 |
| 27 | 80.4 |
| 28 | 80.1 |
| 29 | 80.0 |
| 30 | 80.0 |
| Average | 87.5 |

**Table S3** 8% polyacrylamide gel formulation

| Medicines | Dosage g/L |
| --- | --- |
| TRIS | 10.8 |
| Boric acid | 5.5 |
| EDTA | 0.744 |
| N,N’-Methylenebis | 2.7 |
| Acrylamide | 77.3 |

**Table S4** Sequences of primers used in the study

| Primer ID | Forward Primer | Reverse Primer | Annotation |
| --- | --- | --- | --- |
| SG58（58,707,975） | TGGTTGAAGAAGACCTAGTTGAGG | CCTAGTTCCAAGAAGAGCTCCTT | InDel maker |
| SG59(59,715,567) | CTGATGGAAATATTTAGAAAGAAAGAATGAGC | TAGCAGTGAGTTTTCTTAGACATGCAATTA | InDel maker |
| SG60(60,655,536) | AACAATCATGGCTCAGGAATTTTGCAA | GGGCAATGTGATTTTCTTCTAATTTGG | InDel maker |
| SG60.6(60,656,725) | ATCAAATTGCCCCTGAAGATAGTATCTA | GATTTTACTTTTATACAAAACGCAGCAGC | InDel maker |
| SG61(61,210,879) | CAAACTTGATCTTGTGTGAAGCTTGATT | TTTTCACCCCTATTGATTTTCACTCTC | InDel maker |
| SG61.2(61,232,288 | TAGGTACGTAATGTGAATCTGAACCAA | TAAGATCAATTTTTTACCACGTCTGTGTAAT | InDel maker |
| SG61.4(61,602,870) | GTACGGGCAATCCAAATTAACGC | CAAAATCCTTAGGAGGCCAAGTG | InDel maker |
| SG61.7(61,846,126) | GAGTCATTTAAGTAATTTTAAATCTTGTGCC | GTTAATTTGACTATTCCATAAACAATAACTCTG | InDel maker |
| SG61.8(61,865,087) | ACATGTTGAGATAAAGTCAATAAGAAGATGA | TTGAGTGTTCTTTGGTCGAGGGAA | InDel maker |
| SG62(63,389,272) | CCATTCAAATATTGCAGTAAATTTGAACCTAT | CCGTTAGCATCCATCAGTGGTAA | InDel maker |
| SG63(63,828,438) | AAACTAGTAAGTGATTGCACTACCAAAG | CCTAATATAGTTTTAAGTCAGGAATTAAAATGG | InDel maker |
| SG65(65,291,658) | GTTTGCCTCGTAGATAGCCAAATTGA | GTGCTAATTTGCCATTAGTGCCAAT | InDel maker |
| KL | ATGGGAAGAGGAAGAGTGGAGTTG | TTAAAAAAAAAAAAAGGCAAGAAAAGTAGAAAAAAGAGAGG | CDS amplication |
| SG10  (61,854,327) | TTGGAGTTATAATAGTTTGTGTTTGGAGTGTAG | TCTACGATGCCAAACCCATTCAGACTT | InDel molecular assisted breeding markers |
| *Tan0010544* | TGATCTTCAGAACAAGGAACAAATG | CTCCACCTTCCCATGTTAGTCTTAT | qRT-PCR analysis |
| *Tan0019208.1* | GTAAAAAAGCCTCACAGATACCGTC | TATCTTTAGGCATGATGGTAACACG | qRT-PCR analysis |

**Table S5** Sequence alignment of *TFL*

| Type | Sequence |
| --- | --- |
| CDS Sequence of S1 | ATGGGAAGAGGAAGAGTGGAGTTGAAGAGAATAGAGAACAAGATAAACAGACAAGTGACATTTGCAAAGAGAAGGAATGGACTGTTGAAGAAAGCTTATGAACTTTCTGTTCTTTGTGATGCTGAAGTTGCTCTCATCATCTTCTCTAATCGCGGCAAGCTCTATGAGTTCTGTAGTACTTCCAACATGCTCAAAACACTTCAAAGGTACCAAAAATGCAGTTATGGAGCAGTGGAGGTCACGAAACCGGCTAAAGAGCTCGAGAGTAGCTATAGAGAATACTTGAAGCTGAAAGCTAGATTTGAGTCTCTACAAAGAACTCAGAGAAATCTTCTTGGCGAGGACTTGGGTCCATTGAACTCAAAAGAGCTTGAGCAGCTGGAGCGTCAGCTAGAGTCTTCTTTGAAGCAAGTTAGGTCAACTAAGACACAGTACATGCTGGACCAGCTATCTGATCTTCAGAACAAGGAACAAATGCTGATGGAAACCAACAGAGCTCTGACATTAAAGCTGGAAGAAATAAGTTCAAGAAACAATATAAGACTAACATGGGAAGGTGGAGACCAAAGCATGTCATATGGTCCACAAAATGCACAAACCCAAGGCTTCTTTCAGCCACTAGATTGCAATCCCACTTTGCAAATTGGGTACACTGCAGCAGTATCAGATCAAATCACAGCCACCACTGCTCCAAATCATGCCCAACAAGTCAATGGCTTTCTTCCTGGTTGGATGCTTTGA |
| CDS Sequence of S2 | ATGGGAAGAGGAAGAGTGGAGTTGAAGAGAATAGAGAACAAGATAAACAGACAAGTGACATTTGCAAAGAGAAGGAATGGACTGTTGAAGAAAGCTTATGAACTTTCTGTTCTTTGTGATGCTGAAGTTGCTCTCATCATCTTCTCTAATCGCGGCAAGCTCTATGAGTTCTGTAGTACTTCCAACATGCTCAAAACACTTGAAAGGTACCAAAAATGCAGTTATGGAGCAGTGGAGGTCACGAAACCGGCTAAAGAGCTCGAGAGTAGCTATAGAGAATACTTGAAGCTGAAAGCTAGATTTGAGTCTCTACAAAGAACTCAGAGAAATCTTCTTGGCGAGGACTTGGGTCCATTGAACTCAAAAGAGCTTGAGCAGCTGGAGCGTCAGCTAGAGTCTTCTTTGAAGCAAGTTAGGTCAACTAAGACACAGTACATGCTGGACCAGCTATCTGATCTTCAGAACAAGGAACAAATGCTGATGGAAACCAACAGAGCTCTGACATTAAAGCTGGAAGAAATAAGTTCAAGAAACAATATAAGACTAACATGGGAAGGTGGAGACCAAAGCATGTCATATGGTCCACAAAATGCACAAACCCAAGGCTTCTTTCAGCCACTAGATTGCAATCCCACTTTGCAAATTGGGTACACTGCAGCAGTATCAGATCAAATCACAGCCACCACTGCTCCAAATCATGCCCAACAAGTCAATGGCTTTCTCCCTGGTTGGATGCTTTGA |
| Protein sequence  of S1 | MGRGRVELKRIENKINRQVTFAKRRNGLLKKAYELSVLCDAEVALIIFSNRGKLYEFCSTSNMLKTLQRYQKCSYGAVEVTKPAKELESSYREYLKLKARFESLQRTQRNLLGEDLGPLNSKELEQLERQLESSLKQVRSTKTQYMLDQLSDLQNKEQMLMETNRALTLKLEEISSRNNIRLTWEGGDQSMSYGPQNAQTQGFFQPLDCNPTLQIGYTAAVSDQITATTAPNHAQQVNGFLPGWML |
| Protein sequence  of S2 | MGRGRVELKRIENKINRQVTFAKRRNGLLKKAYELSVLCDAEVALIIFSNRGKLYEFCSTSNMLKTLERYQKCSYGAVEVTKPAKELESSYREYLKLKARFESLQRTQRNLLGEDLGPLNSKELEQLERQLESSLKQVRSTKTQYMLDQLSDLQNKEQMLMETNRALTLKLEEISSRNNIRLTWEGGDQSMSYGPQNAQTQGFFQPLDCNPTLQIGYTAAVSDQITATTAPNHAQQVNGFLPGWML |
| DNA sequence  Of *Tan0010544*  (61851614-61862634) | AAAAAGGGGCGTGGGCAAAGAAGCTGAAAAAAGAAGAGAGAAAAAAAAAAACAGGCTGTTTTATATGAAACTAGAAAGAAGAAGAGAAGAGCCCTAAGCCTGACTTCATATTATTTTTCTGGGTGTGAAGATTGGAAATTCCTTGCAAGTAGTTTTTAAAGCCAAAGAAGAACAAGAGGAAGAGAGAAACAAGACCAAAACATTTTTTTTTGTAATTTTTTGGAGAGAGGGTTTGAAAAAATGTCACTATTTCTCTCTCTACCTCACTAGGGTTTTTTTCCTTTTTTTTTAATTTTTTTTTTGGTTGGATCCCAAAAACAGAGTCTTTGTTTTCACAAAATTAGCTACACTAGAGGGGTACCAACAGAGGAAAGAGGGGATCAAGAAGTACCAGAAGTGAGAAGAAGAAGAAGAAGAAGAAGGAAGAGGAGGAGGAGGAAGAGGGTATTTTAGGGTAAGGAAAAAAAAGAAAGAAAGAAAGAAGGAAATAAAAAGGGAAGGGGAGATTTTGGGTAAAAAAATTGAAGAGGTTTAAAAAGAAAAAAAAAAAGATCAGAAGAGAAATGGGAAGAGGAAGAGTGGAGTTGAAGAGAATAGAGAACAAGATAAACAGACAAGTGACATTTGCAAAGAGAAGGAATGGACTGTTGAAGAAAGCTTATGAACTTTCTGTTCTTTGTGATGCTGAAGTTGCTCTCATCATCTTCTCTAATCGCGGCAAGCTCTATGAGTTCTGTAGTACTTCCAAGTATAGCTCTTCTCTTTGATCTCTTTTATTTTTTATTTTTTATTTTGTTTGTTCGATTGTGGGTTTTTTTTTTTTTTTTGGATTATCTTCTTTTGATCTTGATCTTTCTAGGGTTTCCATTTGTTTATTTTCCAAGAGGAAATAAATAATCCCTTTAGATTTTTAATTCCTTCCTATGTATAATGGGAAGATGGATCCTTTTGAGCTTGTCTTTATTATTATTATTTTTTTTAATGAGGATGTTTAATTCATAGAAAGAAAACACGCAATTCATGTAAGGAAAAAAAAACAACTCAGATTTTTTAAAAGGAGTTTTCAGACATTAACTGACTTTTTTTTTTTTTTTTCCTGATCTTCTGGGTAGGCACAGTTTTTTTTAATTAACTTAATTATAAGCTGACGATTTAGTTCGATGGTTCTTCGTATCATAATTTTAAAACCATTTATTCTTTATTACAGAGAATCAATCATGTCTATTTCTTTCATGGATGGTAATTTAAATAATTTTAGAATTTTGGTAAGCAAAACATAGTGTGTATAAAAAAATAAAAAGAAAAAAAGAGGTTACACTCAGCTGAAACACCTTCAAAAATGGAAATTTCTTACCAAAAAAAAAATAGAGATATGAATAATGCTTTCTTTCTAAATTTGATATCTTGATATAACCCGCTCTATGTCATGTCTTTTCTTGTCTCAGCATGCTCAAAACACTTCAAAGGTACCAAAAATGCAGTTATGGAGCAGTGGAGGTCACGAAACCGGCTAAAGAGCTCGAGGTGAATCATCGATGTCGAGAATATATAAAATGATTAATTTTATTGTAATCCAGTACAAAAAGTCATATTTGATACCCATTTGATTTTTAAAAATTAAATACTACTTTTTTCGTTTTCAAAACATATAGCTTGGTTTTTGGAAAAGATGGATGGAAAGTAGATAATAAAACAAGTAAACCTATAAATTCAGTGTTTATTAGCTAAATTTTAAAAATAAAACTAGATATCAAATGGTTATCAAATGGAGCCGCGGAAAAGTTTTAAAACTGATTTTTTTTTTTTTTTTTGTGTTCATTTTTGCAGAGTAGCTATAGAGAATACTTGAAGCTGAAAGCTAGATTTGAGTCTCTACAAAGAACTCAGAGGTAAATCTTATAAGGATTTGTTTTTGGATGTAATGTATTTTTGTTTAATTATTGATGTTAATTCAGAAAAATTTTCCTACCTATTTAATAAAATATGAATACATCAAGATAATATATGCTCGTATAAACTAGTAGTTACCTTTTAGTTGTTAAGTTTTGCTTAAGGAATTATTATTTAGGCACAAAATTACTTTTAGATATTCAATTAAAATTTCAAACTTAATTCAGTAAGAAAAAAGAAACCAAATAACAAGGGTCCCGTTTGGTAACCATTAGACACAAAATACTATATCTACCTATGAGTTACTATGTTTTCTTATCTATTTTTTACCTATATTTTTGAAAACCAAACTAAACTTTGAAAACTAAAAAAAGTAGTTTTCAAAAACTTGTTTTTTGTTTTTAAAATTTGGCTAGTAATTCAAATGGCTCATGAAGAAAGATGGAAATCATTGTAGAGAAAATTAAGAAATATTGTAGAAAAACAAACATAATTTTCAAAACCCAAAACAAAAAACCATATAGTTATTAAATGGGGCCTAAACATCTATTTCATAACTAATTTATTTCTTAGTTTTCTCTCTTTAAAGATTATACTTGTGTATTTATTTCCTTAGTTTATAACCTCATTTACCCCATTTTAAAAAATGAGAAATATTAATTTTGAACATATCTATTCTTGAAATCTAGCTAAAATTTCATTACTACTTTTGAATAGTGAAATAAAACCAGATAAAAAAAAAGTAGTTGAAAACATATTTCTGTTATCGATATTGTGCTCGCTGTATTATTTTTATTATTTACTATTTTTTTTTTCCTGTTTTCTTATTGACTTATAGAAACGTCTATGAAATTGTAAACAAATGCGGTTCTGAGGATATGTATTCTTGTTTTGTTTTTTGAATTTTGCTTTGAATTCAAAAGTATTTTTAAAAAGTCCATAAAAAGTAATTTTAAAAAGGAAACCACCAAATAAGGAAAAGAGAGATTTAAATAGTTGTCTAAATTCTAAAAAAAAAAAAAGAATATGAAGCCCTGAACCTTGATCAAAGCTTCCTCACCTTAGGTTGAGCCACAATAATTAACCTTAACTGGAATATATGACCCTTTTGATTTGATTAAGGGCCTTCATCTAGATCTCATACAATCAAAACATCTATGAAATAACCCTTATATATGGTCCATTTCTAGAGTTGATTAGCCAATATATATATATTTATATATATATATATATATATTTAAAAAAACTGAGCATTGGAAAAGTATCCAACAATTTTTAATAGGAAGTTTTGGTTCTTTTGTTTCCAAATAGTTGAAAATGGTTGGAAAGGGATCAAAGCCTCATTTTTAGAGTTCTTCATAAAATTATAAATAAATAGATGAGATTTATGTAATTGAAAAGCTAATTGATAAAGGAAAATTTTGTAACCTCTTTATCTCTCTTTTGGGTTGATTTCTCTTATTGAAATGTTTCAGATTTTTTCCTAAAAGAAATAAATTTTAGATAGAAAACCTATGATATTTGTGGATTATCCTAAATAATATGCATAATCTCATAATAATGATTAAAAATAAAGCGTCTAAATTTTAAATGAACATGCTAATATTTCTCTAACATTTCTCTTTAGTTTTTTCCCCTTGTAGGTATATTTGGGAGTTCATTAGGATAAACTTTTTTTAGCAAAATAACTTTAGAGAAGAGGCTAGAGTTTACCCTGTATATAATTGCATCATAAAATGCAAACTTAAGAGATATATTTTAAACTATTAAGAACGATAAGCAAAAGGACTAATTTACCCTTACAAGATAAACCTAAATCAAATATAACCTAATCTAATTTGACAATAAGCACTCCAATAAAAAGAAGAATAGTCATATCACTTTGATGTTTGGTTTATTATTTCTTTTTTATTACTTATATATTTTGACAAAGTTATTTCTGATCGGGTATTTGAGTGAAAAGCAGTCTATTACATATATCTTTCCTATGAGACTCTTCAAATGCCTCTGACTTCTTTTTTTTTTTCCATAATAATTTTGAGGAAAAAGTACTTTCAAACCTTAAATAAGTGGTTTTGGCAGTTGTAGAAAAAATATGGGGTTAGAAATTGATTATTTGTGGGTGTTGTGGCAGAAATCTTCTTGGCGAGGACTTGGGTCCATTGAACTCAAAAGAGCTTGAGCAGCTGGAGCGTCAGCTAGAGTCTTCTTTGAAGCAAGTTAGGTCAACTAAGGTACATTCATCAATATCTAACTTTTTCATTTTTTCTCTTCCTCTCTTTTAAAGTATGATTTTTCAGTGCTAAGTTGAGAGGATAGGAGAGAATCAATACATCTCCGTAGTTCTCTTTAAATCGTTGAGTATATTGTTGAAATTTGATCATTAGACTTGATTGATAATGTAGCGTTTTCATTTCATGTTTCTTATTCTTTGTTTTCAACTTTTAAAAATTAGATATATTTAATGACGTTGTCTGTCTGGTATATGATCCTCATTCTTTACTCGGGCTTATTGAATTGTGTCTACATTTTATAAACAACAAATTTATTATTGTTGTTCTCTTTAGGGTTTTGGATTTTTTTTTTTCGGTTTTTGTTGTATGATCAAGATTTCATTTATTGATTTGCTAATAAAAATTATTTATTGTTTTGTTATATACTTTTGAAAATATTATTGAAATCCTTGGGTAAATTTTAATAACTAGAAAAAATAGCATTTTTGTATGCAAGTTTCTGGTTATGGAAATTTAGCTAAGTGTTTTTAAAAAGTGAAAAACGAATAAAGAAATAGAAATACACTAATTTAATTTTTAAAAACTAAAACGTTATCAACATGCCCGTTAGTATCTTTGTTTTTTATTATTAATTTTCATCGAAGCACTAACAAAGTTGCTTTTTTTAATACATTTTTACTCATAAGTTGCCTATAGTATGTCCAAAATAATAATAATAAATTCAAAATACACAATAATATTAACATTTTCAAATAACTTCTTTTTATCTACCATTTTAGAACTAGTGCTAATTACTTACAGAGAGAAACTTGAGACTTTTGGATCGATCCTTTCCTACTTATAGATTATAAAGAATTCAATTAAATTAAAATTTAGAGATTTCAAAATATATCTTTTTGGATTTCAAGTCATTTCGTCTCTTAATTTTTTTTGTTTGTTTTTTTCTCTCCATTAAAAACCAGCATTAAAAGATTTTTTTTTAATTCAACATTTAGAAGAACGAAAGATTCGAATGTATAACCTCTCGGTCATATACTTATATTTTATGATTAACTATTAGGAATATAGAAACTAGTTAAAACTAAATAATGGAGATGATTTGCAGATTTAATTTCCAGCGTGGTAATTTTCATATGAATTTCTCTTTTTCAGTTGCCAGGAGCATGAGCTTTCCAATCAAATAAATTTTTAATGCCTAATTTCTTATTTCAATGCGTACATTAATTGTATTTTTAGCGAATAATTAATTTAAAATCTTTTACACTCCAATATCTTATAGAAAAAAAAAACCTTGGAAGTCTTTGAAGTGTATTTTTTCATAAAATAAAGTTGGAGTTCTGTTAAAAAGTAAAAGATAGATATTGAATGGACCAATATTCTCAGTCTTTAAACAAGAATTTGAACAAATTAATAACCTAAATAACCTAAACTAACTCAACTTAAATATTTGATTGTTAGGTTCAATCATCTAGTTCGAGTCAAATAAAGTAATTTAACAATTAAGAAAAAGTCTCTTAACCCAATTCAATCCAATTTATAATAAAAAACAAGTGTCAATATGACGATCCAAATGCATGTTTAACTTCTTTTTCTCCTCCCCGAAATAAATTTAAGAAAAGAAATTTAATTCCAGCATAGAAAGAGAAACCATATGATTTTTTTTTTTTAAAAAAAATATTGTTTTATGTGGAATTATTTTTTTTTCTTTAAAATTAGTATTTTCTTATATTGATCTTAAGGACAAATTATTCAATGAGACCGTCCTGATGCAAAACTGCTAATCATATTATGATGGTCTCATCGAATAAATTCAAAATGGAAGCACTTTCTTACTAACATGTGTCTACGAATTGACAATTTTATTTCAATTCTCATCCTCATTCTTATTTCTAGACTTTTATCTCAATGCAAATTATTCTAATATATATTTTTTCCTTTTTTGAGGATAATACAAATATCTCTTTTATTACCATATATAAAACGCCACACCTTTATTTTGAAATATCTAAGCTTCATTTTTCTATTCTCCCATCCAAATATTCCCTTCTGTTCTCTAGTTTTCAAACTCAAAATAACTTTATTTTTCCAATCTTCCACCCCCAAATGTTGATGATATACTAAAAAAAATAATTTTATTCTTAAGCATTTTATCCTTATATATTTCTCTTTCTTTAAATTTTTTAATTTTTATTCTGAGGTTGGCATAGAATATCTCTATACTTTATTTGATGTCTTCAGTTTCCATTTCATTTTTTGTTGTAATTTCTCATAAGAAGAGTGTATCTTGCCGTCCTAAGATTAGTTCAACTAGTTAGAGTTATCATTGTTCAAAAGATTAAAGATTTAAATCTTCACTTCTACTAGTTGAACTAATATATATATATACCATCTTCTATTGATCTTTTTTTTCTTTTCTTACTCTAATTTTAGACTTTTGATTCTTTTGCTGAATGTTCAAGATCTTTTAATTTATAACAAAGTTTTTATTCAATTTGAAACATCATAAAAAGGCATGTTTATAGACTTGTTGCTCTACAACAATCGGAGGTGGAGAGATTCAAATCTCTGACCTTTTGGTCGAAGTTACATGCCAATTACTATACCGAGCTAAGCTCACTTTGGCACGGGTTGTTGTACTTGTTTGTGTTAACAGTAAACCATCAGCCTGATTTTTTAATTTTTTAATTTTTATAATCATAAATCTAGTAAAATTGAAATAAAATGTCGTAAAAATAACATTAACAGATATGTAATCCTCACTTAAGTTGTTTTTTTTTTTTTTTTTTTTTAATTTAATACATAACTTCTCATTCTGAATTATCTTTGTCAACATTTAATAAGAATAATCATCTCATCTAAGTAGAAATATGTATTCATCATTCTATTAGAAATATAAATTAAATTCCGATTCCAATCCTTTTAAACTTTGCTACTTCTTCCATTTAACATTTTGGTCTCTATGTTTTCGTTTCTTTTAGATTAGCCCCCATATGTTATATGAACATATAAATTTAATCCCAACACTATCACTAAATTATAAATAGCAATACAAAGTTATTTAGAAATAAAAATTGTAAATTTTGTTCTGCATGTGTCTTGTCCCAAACATTATCCATGTGGCTGTGCTATGTAGACAACTATTGAAGCCTTCACTTTTGTACTTTCGAAATGGCTCTCTCAGTTGCTCTGCTTCTTAATTAATACCCATTTATTAACTTCCACCATTCTCTGGCTTTTAACCATAGTATTAAAGCCGGTAAAGATAAAGACGTTTATGGTTAAAAAAAATACTTTTAATTATCGTATTCAAAAACTAATTCGAAACATGTTTTTAATTATTCAAAATCAAATTGAAAAGTGTAAAATCAAATGGTTGGCAAATTTTGAACGATTTAAAAGCATGTGTTAGAGTCATTTTGAATTTGACAAAAGTGATTTTGACCATCTCAAAATCACTCCCAAATATGTCTTTAATAAGTTATGAAATTTGATGTCGTTATACATTTCGTGCTTGGATTATGTAACTTGTGTGTTTGGTTTTTGATCGTTTATGATTTTAAAAGGATAGGATATGGTATGATAAATGTTTTGTAGCACATGAAATTAATGTAAACCTGTAGCACTGAGCAAGGATATTCATATGTTGAAATTTAGGCATGATTTAACCTTGGATTATACAAGTCCAGCCCATTGGGATTTCTTTATATTTTATAATTTTGTTTTGAGTTCAACAATATTCGAGTGTGGGAGATTCGACCTTTTGATTTTTTGGTAAAAAATTGATCTAAATTACCACTAAATTATGCTTACTCTAGCTTCTTAATGGACTTCTTTATGTTTCCATTACAGGCTTGTTTTTATCGTTTTTAGCATATTCTTATCTTGGAATATAGGTTTGATTTCTTAGCCTTGCGTTATATGATAAAATACTTTTCCGGATGGCTATTTCTATGGATCTTTATAATTATCCCTTATGTTTGATAAGCTATTAGTTAGTATTTGAGGCGACTCCTCTTGTTGAGCTGTTTTCTATGGGTCCTTATGTACTTTTTTTTTTTCTCTCAATGAAACCGAGTTTCTAATCAAAGAAATCTTACCAGTCTAGTCTACGATGCCAAACCCATTCAGACTTCTTATATTATCTCTAAATGTCTGTTTAGGGCACATACTATAATAACCTGTGAAATATTCTACACTCCAAACACAAACTATTATAACTCCAAACTATACTACTTTTCATTTTAAATAATATTATATGATTCTACAGATTATAATAACCACAGACTATTATAACTCTTGTTATTATAACCCACTTAGCGTTCCAAACAACGCTTAAGAAACTTAGGTTGATGTGCTTCGTGACATTAACAAATCCATAAGGTAGAATAAGCAGATTTCTTATACCAAACACTTTTTTTATGTGTTTGAAAAACCTTTGGTCAGTGACCAGTTGTCTGATTAGCTCATAATGTGTGTAATTAGTTAATTATTAACAAAATGTGGGAAATGGGAATTTGCAGACACAGTACATGCTGGACCAGCTATCTGATCTTCAGAACAAGGTAGGCTTGACTTGATATCAAGTTTTAATTTCTGTTGAATTGTCATTAATATGCTAAGTTTTTTTTTTTTTTTCTCTTTTTTTCAAGTACAGGAACAAATGCTGATGGAAACCAACAGAGCTCTGACATTAAAGGTAATCCACATTTCTTTCTACACACACAAGAAAGATCTTAAAATTACTGTTTCTCAAACATTGGAAGTACAAAAAAAAAAAAAAAGTTGTATTCTTGCATGTTGGAAAAAGATTAAATTTACTGTTGCCTGCTCATTTAAAAGCATTCTTGAATATGCTTATAAAGTACTTAAGTGAAAAGTGCTCGATAACAAATCATTTAACTCGTTTTGTTCTAAATTTAGTACATCTGTTTTGAAGTATTTTTTATATATTCTACCATTACTTTTTTATAGAACAAGTTTTAGGTATTTCAAGCTACTCTCAAACATGATAATTCGGAGTGGAAAGTGTCTTCTTAAGGTTTTTGTATCTAATAGTTTAATATGTTGTTAAATCAGGAACAATCGAGTTAGAATTGCTTCAAAGAGTGTAGCTTTTAGAGTAGGAAACCTTTTTAGATAGCAATCTTCTCTAGTTTTTTTGGGCAAAATATATTTTTGGTCTCCGAGATTTGGAGTCGGTGTCTATTTGGTCTTTAAAGTTTCAAAATGAACCGTTTAGTCTTTAAAATTTGGAAAATACTTTTAATTGGTTCCTAAAGTTAGTTAACTAACGAAAATATGATGTGATGTGGCATGTTAAAGTGACAATTTTTAGAATGTTAAGATTTATATTAATTTTACCTTATGTATCCATTTTGTAAAATCCAAATCGCAAAAAGGAAAACTTAATTTTACTTTTCCTTCATATGCTGTTCCTTTATTCTTTTTTTTTTTCTCTTCATCTTCCTCTATCTCCTTTTTTCCTGCTTCAGATTTTTGTTTGTTTACTCTGGAACTTTTGTTATGGAGTTGATTTTCTATTTATTTTTTCTTTTCTCGATTTGGGTTTTCAGTTTTCGACTCTGTAATCTCGTATTTCCTTTGTTTTCTCTCTCTTTTGATACTTTGAGAGCCTTGTTGGTTTTCTCTGTAATATTCAGTTCTCAACTCCTCAATCTTATATTTCTCTTGTTTCCCCTCATCTTTTGCTCTTCTTCTTCCTCTATCTCTATTGTAAAAATATTATTTTAAATAAAAGAAGAGAAGAGGGATCTTTAACATGCCACTTTAACATAAACATTGTCACTTTAACATGCCACGTCATTCTTCTGTTAGTTAACTAACTTTAGCGACCATTTCAAACTATTTTCCAAATATCAAGAGTTGAAATGTTTATTTTGAAACTTCAGAGACCAAATATCGATCTCTTAACAGGGAGGATCAAAAGTGTATTTTATCATTTTTTTTTTCTAAAAATCATGTTCGATTATTGATTCTTTCCTTTTCCTAGGGGGGCATTATAATCTACCCTTAGAAAAATGTCTCTAAAGTCCCATGTAGTCAAGATTTCAAACAAAGTTAAGAAGAAATAACAAAACTAAAACTAAAATCTTTATTGATACAAATTGCTAAGAGACAACAAATCCCTTTTAATATGTTGGTTTAGTATTTGGATTGGTTGATCAGCTGGAAGAAATAAGTTCAAGAAACAATATAAGACTAACATGGGAAGGTGGAGACCAAAGCATGTCATATGGTCCACAAAATGCACAAACCCAAGGCTTCTTTCAGCCACTAGATTGCAATCCCACTTTGCAAATTGGGTTAGTTTTTTTTTTTTTTTTTTTTAATTCTTTTTTTCCCTTTTCTATAAGCAAATGAAATTTAATGAATTTTTTTTTTGGGATAATTTGTTTTTTATTTTTGTGGTGGGTTTTAATCAGGTACACTGCAGCAGTATCAGATCAAATCACAGCCACCACTGCTCCAAATCATGCCCAACAAGTCAATGGCTTTCTTCCTGGTTGGATGCTTTGAAAACCAAAACTACCTCTCTTTTTTCTACTTTTCTTGCCTTTTTTTTTTTTTAATTGCACTATGCTACCAACAGTTTGCAAAATTGTTAATAAGCATTATATTTTACTTTTATTTTAGACATTAAAAGAAAACCCCAAAATTTGTCTTGTGGAGATGTCAATGGAAAAGTGGAAATTAAAAAAAAAAAATTGAAAAAACAAAGGGTTTCCACAGACAAATTTGAAGGACTTGTATCCAGAACATATCATATTCACATGTATTTTTAAATTTTAAGACGCTTTAGATTTTGTATTGGATTTTGATTTCTATTTCATGGTAATGTGCTTTTGTATATTTCGAAATAGAGGTGGAATTATTTTA |

**Table S6** Amino acid sequence information contained in phylogenetic analysis

| Name | Sequence |
| --- | --- |
| XP_022155022.1(Momordica charantia) | mgrgrvelkrienkinrqvtfakrrngllkkayelsvlcdaevaliifsnrgklyefcstsnmlktleryqkcsygavevtkpakelessyreylklksrfeslqrtqrnllgedlgplnskeleqlerqlesslkqvrstktqymldqlsdlqnkeqmlietnraltlkleeissrnnirltwdggdqsmsygpqnaqtqgffqpldcnptlqigytaavsdqmtastapthaqqvngflpgwml |
| NP_001267667.1(Cucumis sativus) | mgrgrvelkrienkinrqvtfakrrngllkkayelsvlcdaevaliifsnrgklyefcstsnmlktleryqkcsygavevtkpakelessyreylklksrfeslqrtqrnllgedlgplnskeleqlerqlvsslkqvrstktqymldqlsdlqnkeqmlietnralqikleeissrnnirltwdggdqsmsygpqnaqtqgffqplecnptlqigytsavsdqitstttpthaqqvngfLpgwml |
| XP_022955902.1(Cucurbita moschata) | mgrgrvelkrienkinrqvtfakrrngllkkayelsvlcdaevaliifsnrgklyefcstsnmlktleryqkcsygavevtkpakelessyreylklkarfeslqttqrnllgedlgplnskeleqlerqlesslkqvrstktqymldqlsdlqnkeqmlietnralqikleeissrnnirltweagdqsmsyapqdaqtqgffqaldcnptlqigytsavsdqitattapthaqqvngfLpgwml |
| XP_022998764.1(Cucurbita maxima) | mgrgrvelkrienkinrqvtfakrrsgllkkayelsvlcdaevaliifsnrgklyefcstsnmlktleryqkcsygavevakpakelessyreylklkarfeslqrtqrnllgeelgplnskeleqlerqlesslkqvrstktqymldqlsdlqnkeqmlietnrsltlkleeissrnnirltweevdqgmsyapqnaqtqgffqpldcnptlqigysaavsdqitattapnhaqqvnaflpgwml |
| XP_008464576.2(Cucumis melo) | Mgrgrvelkrienkinrqvtfakrrngllkkayelsvlcdaevaliifsnrgklyefcstsnmlktleryqkcsygavevtkpakelessyreyvklksrfeslqrtqrnllgedlgplnskeleqlerqlesslkqvrstktqymldqlsdlqnkeqmlietnralqmkleeissrnnirhpwdggdqsmsygtqnaqtqgffqpldcnptlqigytsavsdqitstttpthaqqvngflpgwml |
| XP_023527245.1(Cucurbita pepo subsp. Pepo) | mgrgrvelkrienkinrqvtfakrrngllkkayelsvlcdaevaliifsnrgklyefcstsnmlktleryqkcsygavevakpakelessyreylklkarfeslqttqrnllgedlgplnskeleqlerqlesslkqvrstktqymldqlsdlqnkeqmlietnralqikleeissrnnirltweagdqsmsyapqdaqtqgffqaldcnptlqigsdfrytsavsdqitattapthaqqvngflpgwml |
| AXR86364.1 (Kerria japonica) | mgrgrvelkrienkinrqvtfakrrngllkkayelsilcdaevaliifsnrgklyefcssssilktleryqkcsygqvevtkpakelessyreylklkgrfeslqrtqrnllgedlgplntkeleqlerqleaslkqvrstktqymldqlsdlqnkeqmlieanrdltikldeistrsqfRqswgghgdqgmaygthnaqsqgffqpldcnptlqigystvgseqmnatthaqqvngfipgwml |
| XP_006585806.1(Glycine max) | mgrgrvelkrienkinrqvtfakrrngllkkayelsvlcdaevaliifsnrgklyefcssssmlktleryqkcsygavevtkpakelessyreylklkarfeslqrtqrnllgedlgplntkelehlerqldsslkqvrstktqfmldqlsdlqtkeqmlveanrsltvkleeinsrnhyRqsweagdqsmpygggpqnshshqgffqplecnptlqigpdyryndvasdqitattqpqqvsgfipgwml |
| NP_186880.1 (Arabidopsis thaliana) | mgrgrvelkrienkinrqvtfakrrngllkkayelsvlcdaevslivfsnrgklyefcstsnmlktleryqkcsygsievnnkpakelensyreylklkgryenlqrqqrnllgedlgplnskeleqlerqldgslkqvrciktqymldqlsdlqgkehilldanralsmkledmigvrhHhigggweggdqqniayghpqahsqglyqslecdptlqigyshpvcseqmavtvqgqsqqgngyipgwml |
| KAF3944841.1 (Castanea mollissima) | mgrgrvelkrienkinrqvtfakrrngllkkayelsvlcdaevaliifsnrgklyefcstssmlktleryqkcsygavevnkpgkeleisyreylklkarfeslqrtqrnllgedlgplntndlerlerqldsslkqvrstktqymldqlsdlqnkehllveanraltikldeissrnnlRqsweggeqsmsygpqnahsqsffqpldcnptlqigynasgsdqqlsgtthaqqvngfipgwml |
| XP_007032865.1(Theobroma cacao) | mgrgrvelkrienkinrqvtfakrrngllkkayelsvlcdaevaliifsnrgklyefcssasmlktleryqkcsygavevskpakelessyreylklkaryealqrtqrnllgedlgplnskeleqlerqletslkhvrstktqylldqlsdlqnkeqmlmeanralsikldeisarnqfrasweggeqsvpygnqqaqsqglfqplecnptlqigynpvasdqiaatthaqqvngfipgwml |
| XP_042975042.1(Carya illinoinensis) | mgrgrvelkrienkinrqvtfakrrngllkkayelsvlcdaevaliifssrgklyefcssssmlktleryqkcsygavevnkpakelessyreylrlkarfeslqqtqrnllgedlgplnsndleklerqlesslkqvrsiktqymldqlsdlqnkeqllldsnrtltikldeicsrnhlrqtwdqggeqsisygpqnaqiqgffqpldcnptlqigynasgsdqinatthaqqvngfipgwml |
| XP_020205009.1(Cajanus cajan) | mgrgrvelkrienkinrqvtfakrrngllkkayelsvlcdaevaliifsnrgklyefcssssmlktldryqkcsygavevskpakelessyreylklktrfeslqrtqrnllgedlgplntkdleqlerqldsslkqvrstktqfmldqladlqnkeqmlveanrsltmklneinsrnhyrqsweagdqsmayggpqnahsqgffqplecnptlqigyngvasdqitatsqaqqvngfipgwml |
| Solyc02g089200.4 (Solanum lycopersicum L) | MGRGRVELKRIENKINRQVTFAKRRNGLLKKAYELSVLCDAEVALLVFSNRGKLYEFCSTNNMLKTLDRYQKCSYGTLEVNRSIKDNEQSSYREYLKLKAKYESLQRYQRHLLGDELGPLTIDDLEHLEVQLDTSLKHIRSTRTQMMLDQLSDLQTKEKLWNEANKVLERKMEEIYAENNMQQAWGGGEQSLNYGQQQHPQSQGFFQPLECNSSLQIGYDPITTSSQITAVTNAQNVNGMIPGWML |
| RYR32938.1 (Arachis hypogaea) | MGRGRVELKRIENKINRQVTFAKRRNGLLKKAYELSVLCDAEVALIIFSNRGKLYEFCSSNSMLKTLERYQKCSYGAVEVNKPAKELENSYREYLKLKARFESLQRTQRNLLGEDLGPLGTKELEQLERQLDTSLRQVRSTKTQFMDQLADLQNKEQILVEANRSLTMKLEEINSRNQYRQPWEAGEQSMQYGTQNAHSQGFFQPLECNPTLQIGYNPVASDQITATTQAQQVNGFVPGWML |
| Smechr0202837 (Solanum melongena L) | MGRGRVELKRIENKINRQVTFAKRRNGLLKKAYELSVLCDAEVALLIFSNRGKLYEFCSSNNMLKTLERYQKCSYGTLEVNRSIKDNEQSSYREYMKLKAKYESLQRYQRHLLGDELGPLTIDDLEHLEAQLDTSLKHIRSTRTQMMLDQLSDLQTKEKLWNEANKVLERKQMEEIYAENNLHQPWGGGEQSLTYGQQQHPQSQGFFQPLDCNSSLQIGYDPITTSSQITAVTNAQNVNGMIPGWML |
| OsNip_08g0531700  (Oryza sativa L) | MGRGRVELKRIENKINRQVTFAKRRNGLLKKAYELSVLCDAEVALIIFSNRGKLYEFCSTQSMTKTLEKYQKCSYAGPETAVQNRESEQLKASRNEYLKLKARVENLQRTQRNLLGEDLDSLGIKELESLEKQLDSSLKHVRTTRTKHLVDQLTELQRKEQMVSEANRCLRRKLEESNHVRGQQVWEQGCNLIGYERQPEVQQPLHGGNGFFHPLDAAGEPTLQIGYPAEHHEAMNSACMNTYMPPWLP |

**Table S7** Phenotype-genotype identification of Fruit length in 27 snake gourd accessions Resources

| Number | Variety Name | Fruit Length | Genotype |
| --- | --- | --- | --- |
| 1 | S1 | Long | Long |
| 2 | S3 | Long | Long |
| 3 | S9 | Long | Long |
| 4 | S10 | Long | Long |
| 5 | S11 | Long | Long |
| 6 | S13 | Long | Long |
| 7 | S16 | Long | Long |
| 8 | S17 | Long | Long |
| 9 | S19 | Long | Long |
| 10 | S2 | Short | Short |
| 11 | S4 | Short | Short |
| 12 | S5 | Short | Short |
| 13 | S6 | Short | Short |
| 14 | S7 | Short | Short |
| 15 | S12 | Short | Short |
| 16 | S14 | Short | Short |
| 17 | S15 | Short | Short |
| 18 | S18 | Short | Short |
| 19 | S19 | Short | Short |
| 20 | S20 | Short | Short |
| 21 | S21 | Short | Short |
| 22 | S22 | Short | Short |
| 23 | S23 | Short | Short |
| 24 | S24 | Short | Short |
| 25 | S25 | Short | Short |
| 26 | S26 | Long | Long and Short |
| 27 | S27 | Long | Long and Short |

| Semple mane | Reads number | Reads length | Tolal base | Q20 content(%) | Q30 content(%) | GC content(%) |
| --- | --- | --- | --- | --- | --- | --- |
| SA | 238457730 | 150 | 35768659500 | 98 | 94 | 38 |
| SB | 221257766 | 150 | 33188664900 | 97 | 93 | 37 |
| Sd | 234769954 | 150 | 35215493100 | 97 | 93 | 38 |
| Sh | 224787160 | 150 | 33718074000 | 97 | 92 | 38 |

**Table S8** Statistical table of sequencing data of each sample

**Table S9** Phenotypes of recombinant exchange plants

| ID | | FL(cm) | |
| --- | --- | --- | --- |
| F_2_-19-1 | 25.0±3.2 | |  |
| F_2_-25-76 | | 23.6±2.9 | |
| F_2_-5-16 | | 28.4±3.0 | |
| F_2_-21-76 | | 74.4±15.3 | |
| F_2_-27-65 | | 83.3±17.5 | |

**Table S10** *TFL* determines the interval

| Name | Chromosome | QTL Interval | ID | Gene annotation | ΔSNP-index value |
| --- | --- | --- | --- | --- | --- |
| TFL | Chr4 | 61,846,126 bp-61,865,087 bp | Tan0010544 | MADS-box transcription factor family | 0.71 |

**Table S11** Amino acid sequence information contained in the phylogenetic analysis of the *Arabidopsis thaliana* MADS-box

| Name | Sequence |
| --- | --- |
| AT1G22130.1 | MGRVKLEIKRIENTTNRQVTFSKRRNGLIKKAYELSILCDIDIALIMFSPSDRLSLFSGKTRIEDVFSRFINLPKQERESALYFPDQNRRPDIQNKECLLRILQQLKTENDIALQVTNPAAINSDVEELEHEVCRLQQQLQMAEEELRRYEPDPIRFTTMEEYEVSEKQLLDTLTHVVQRRDHLMSNHLSSYEASTMQPNIGGPFVNDVVEGWLPENGTNQTHLFDASAHSNQLRELSSAMYEPLLQGSSSSSNQNNMSECHVTNHNGEMFPEWAQAYSSSALFASMQQQHEGVGPSIEEMMPAQQSDIPGVTAETQVDHEVSDYETKVPQLSSQ |
| AT1G24260.1 | MGRGRVELKRIENKINRQVTFAKRRNGLLKKAYELSVLCDAEVALIIFSNRGKLYEFCSSSSMLRTLERYQKCNYGAPEPNVPSREALAELSSQQEYLKLKERYDALQRTQRNLLGEDLGPLSTKELESLERQLDSSLKQIRALRTQFMLDQLNDLQSKERMLTETNKTLRLRLADGYQMPLQLNPNQEEVDHYGRHHHQQQQHSQAFFQPLECEPILQIGYQGQQDGMGAGPSVNNYMLGWLPYDTNSI |
| AT1G24260.2 | MGRGRVELKRIENKINRQVTFAKRRNGLLKKAYELSVLCDAEVALIIFSNRGKLYEFCSSSSMLRTLERYQKCNYGAPEPNVPSREALAVELSSQQEYLKLKERYDALQRTQRNLLGEDLGPLSTKELESLERQLDSSLKQIRALRTQFMLDQLNDLQSKERMLTETNKTLRLRLADGYQMPLQLNPNQEEVDHYGRHHHQQQQHSQAFFQPLECEPILQIGYQGQQDGMGAGPSVNNYMLGWLPYDTNSI |
| AT1G24260.3 | MGRGRVELKRIENKINRQVTFAKRRNGLLKKAYELSVLCDAEVALIIFSNRGKLYEFCSSSSMLRTLERYQKCNYGAPEPNVPSREALAVELSSQQEYLKLKERYDALQRTQRNLLGEDLGPLSTKELESLERQLDSSLKQIRALRTQFMLDQLNDLQSKLADGYQMPLQLNPNQEEVDHYGRHHHQQQQHSQAFFQPLECEPILQIGYQGQQDGMGAGPSVNNYMLGWLPYDTNSI |
| AT1G26310.1 | MGRGRVELKRIENKINRQVTFSKRRTGLLKKAQEISVLCDAEVSLIVFSHKGKLFEYSSESCMEKVLERYERYSYAERQLIAPDSHVNAQTNWSMEYSRLKAKIELLERNQRHYLGEELEPMSLKDLQNLEQQLETALKHIRSRKNQLMNESLNHLQRKEKEIQEENSMLTKQIKERENILRTKQTQCEQLNRSVDDVPQPQPFQHPHLYMIAHQTSPFLNMGGLYQEEDQTAMRRNNLDLTLEPIYNYLGCYAA |
| AT1G31140.1 | MRKGKRVIKKIEEKIKRQVTFAKRKKSLIKKAYELSVLCDVHLGLIIFSHSNRLYDFCSNSTSMENLIMRYQKEKEGQTTAEHSFHSCSDCVKTKESMMREIENLKLNLQLYDGHGLNLLTYDELLSFELHLESSLQHARARKSEFMHQQQQQQTDQKLKGKEKGQGSSWEQLMWQAERQMMTCQRQKDPAPANEGGVPFLRWGTTHRRSSPP |
| AT1G01530.1 | MARKNLGRRKIELVKMTNESNLQVTFSKRRSGLFKKGSELCTLCDAEIAIIVFSPSGKAYSFGHPNVNKLLDHSLGRVIRHNNTNFAESRTKLRIQMLNESLTEVMAEKEKEQETKQSIVQNERENKDAEKWWRNSPTELNLAQSTSMKCDLEALKKEVDEKVAQLHHRNLNFYVGSSSNVAAPAAVSGGNISTNHGFFDQNGNSTSAPTLPFGFNVMNRTPAGYNSYQLQNQEVKQVHPQYWARYY |
| AT1G17310.1 | MKDLFMEGERETSSMTCLTPKDSVQSPNMLVRQPKKETTTQTPKTTRGRQKIEIKKIEEETKRQVTFSKRRRGLFKKSAELSVLTGAKIAVITFSKCDRIYRFGHVDALIDKYLRKSPVKLEGYSGDNAADEESRRPWWERPVESVPEEELEEYMAALSMLRENIGKKIVAMGNDRTVDMVPAWPINVMGWKPTMDMQKLENLTDGVNRCRVGQNGD |
| AT1G18750.1 | MGRVKLKIKRLESTSNRQVTYTKRKNGILKKAKELSILCDIDIVLLMFSPTGRATAFHGEHSCIEEVISKFAQLTPQERTKRKLESLEALKKTFKKLDHDVNIHDFLGARNQTIEGLSNQVAIYQAQLMECHRRLSCWTNIDRIENTEHLDLLEESLRKSIERIQIHKEHYRKNQLLPIECATTQFHSGIQLPMAMGGNSSMQEAHSMSWLPDNDHQQTILPGDSSFLPHREMDGSIPVYSSCFFESTKPEDQICSNPGQQFEQLEQQGNGCLGLQQLGEEYSYPTPFGTTLGMEEDQEKKIKSEMELNNLQQQQQQQQQQQQQDPSMYDPMANNNGGCFQIPHDQSMFVNDHHHHHHHHHQNWVPDSMFGQTSYNQVCVFTPPLELSR |
| AT1G22590.2 | MGRRKVTHQLISDNATRRVTFRKRKDGLLKKIYELTVLCGLPACAIIYSEYKDGPELWPNLNEVRSILNRLSELPVEKQTKYMMDQKDLMNKMIQDAEKKLEKEKMHTRAMKLGLMAGSNDLITDTDCSEELARAADVVDKKLKAIRERIKAVEAGAPIIKRD |
| AT1G28450.1 | MNPKKTKGKQKINIKKIEKDEDRSVTLSKRLNAIYTMIIELSILCGVEVAFIGYSCSGKPYTFGSPSFQAVVERFLNGEASSSSSSSLQRSVKNAHKQAKIQELCKRYNRLVEELKVDEVKVKKAAALAETRAVNKDAWWKADPNDVKDHEKAKKMMEKYQELKEKLREEVALRIKRGHDENNNK |
| AT1G28460.1 | MNPKKTKGKQKINIKKIEKDEGRSVTFSKRLNGIYTKISELSILCGVEVAFIGYSCSGKPYTFGSPSFQAVAERFLNGDASSSSSSSLVMNAHKQAKIQELCKKYNRLVEELKVDEVKVKKAAALAETRVVNKDVWWKVDPNDVKDHEKAKKMMEKYQELYDKLCEQAASRIKRGHDENNNK |
| AT1G28460.1 | MNPKKTKGKQKINIKKIEKDEGRSVTFSKRLNGIYTKISELSILCGVEVAFIGYSCSGKPYTFGSPSFQAVAERFLNGDASSSSSSSLVMNAHKQAKIQELCKKYNRLVEELKVDEVKVKKAAALAETRVVNKDVWWKVDPNDVKDHEKAKKMMEKYQELYDKLCEQAASRIKRGHDENNNK |

Supplementary Fig 1 Phylogenetic tree of the TFL and Arabidopsis MADS-box

**
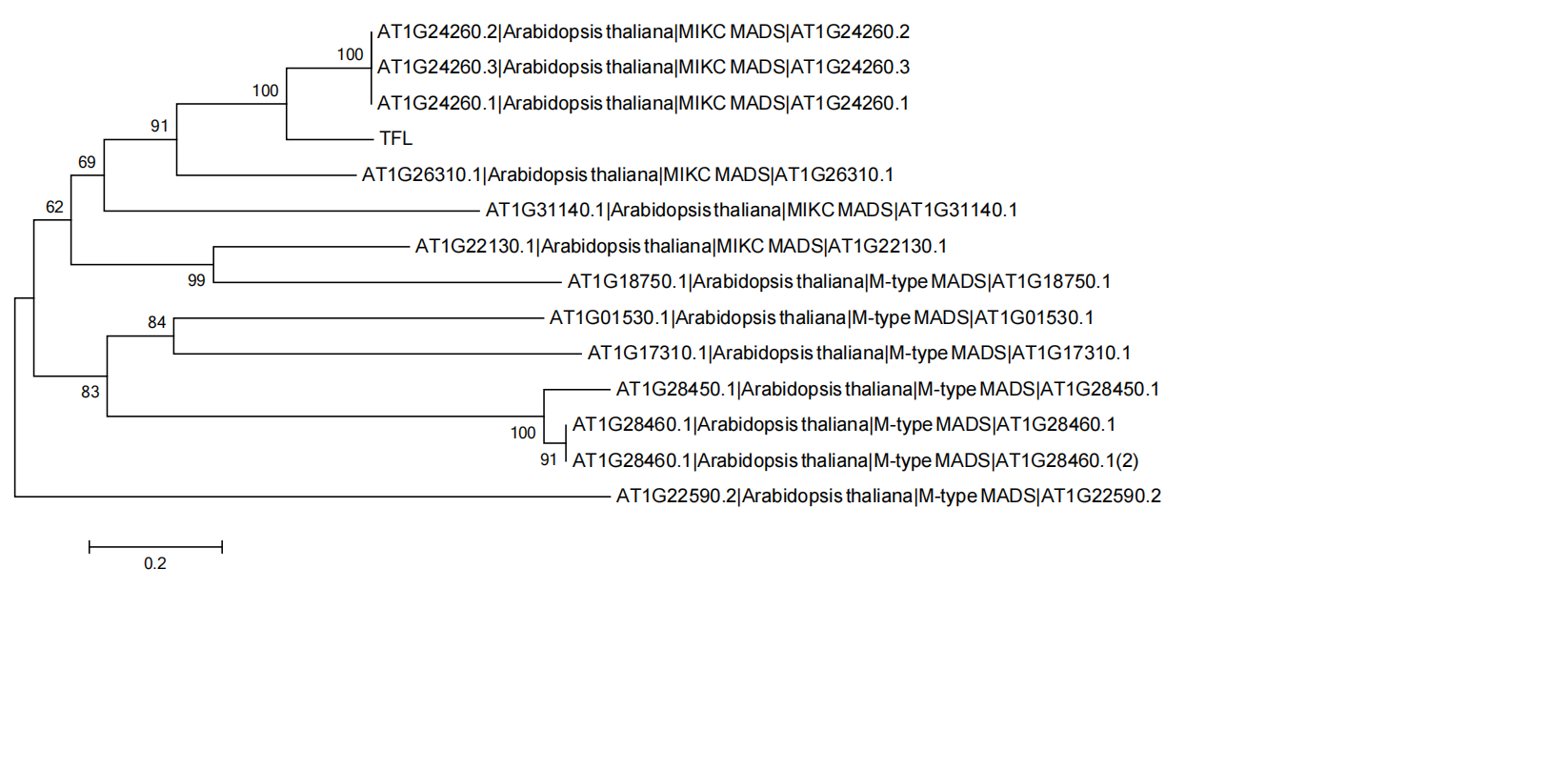
**
